# Structure analysis in an octocopter using piezoelectric sensors and machine learning

**DOI:** 10.1038/s41598-025-17265-x

**Published:** 2025-08-28

**Authors:** Andrzej Koszewnik, Bartłomiej Ambrożkiewicz, Daniel Ołdziej, Pawel Dzienis, Mateusz Pieciul, Arkadiusz Syta, Jacek Zaburko, Ghada Bouattour, Justinas Gargasas, Kristina Baziene

**Affiliations:** 1 Bialystok University of Technology, Wiejska Street 45C, Bialystok, 15-351 Poland; 2 Lublin University of Technology, Nadbystrzycka 38, Lublin, 20-618 Poland; 3 Leuphana University Lüneburg, Universitätsallee 1, Lüneburg, 21335 Germany; 4 Vilnius Gediminas Technical University, Saulėtekio al. 11, Vilnius, LT-10223 Lithuania

**Keywords:** Data, Piezo-composite elements, Statistical learning, Feature selection, Unmanned Aerial Vehicle, Structural health monitoring, Failure detection and identification, Mechanical engineering, Aerospace engineering

## Abstract

This study presents a novel diagnostic methodology for assessing drive system damage and its propagation in an unmanned aerial vehicle (UAV) using piezoelectric sensors mounted on each arm of the drone. In contrast to existing studies that focus solely on fault localization, this work investigates the spatial propagation of structural responses to localized motor faults under varying operating conditions. By varying the PWM control signal duty cycle on one motor, different degrees of damage (from 20% to 80%) were simulated. Voltage signals were recorded on each arm of the drone to identify damage and to optimize the number and placement of the sensors. Statistical features extracted in both the time and frequency domains were calculated within sliding time windows. These features (e.g., mean, variance, spectral skewness, spectral kurtosis) from voltage time-series were used as input data for machine learning models (e.g., Random Forest and K-Nearest Neighbors), which are widely applied in the diagnostics of rotary systems for binary classification problems (distinguishing between intact and damaged states of varying damage level). The highest classification accuracy was achieved for the arm where the electric motor failure was induced (from 93% to 94% depending on the degree of damage), while the lowest accuracy was obtained for the opposite arm (from 50% to 57% depending on the degree of damage). It was found that diagnostic accuracy increases when frequency-domain features of the signals are used, particularly for the opposite arms. The proposed methodology provides valuable insights into the structural behavior of the drone in both ground and flight conditions, illustrating the propagation of local damage to other components. The results contribute to the development of robust diagnostic techniques for health monitoring and structural reliability assessment of unmanned aerial vehicles (UAVs).

## Introduction

The utilization of unmanned aerial vehicles (UAVs) continues to expand annually, accompanied by the diversification of their applications. In comprehensive reviews^1,2^ key domains in which UAVs are identified, including infrastructure^3^, agriculture^4^, transportation^5^, security^6^, media and entertainment^7^, telecommunications^8^, and mining^9^. Furthermore, these reviews highlight specific applications where UAVs demonstrate significant utility, such as search and rescue operations^10^, remote sensing^11^, construction and infrastructure inspections^12^, and precision agriculture^13^.

Technological advancements in UAV systems have been marked by notable progress in sensor technology^14^, onboard computational capabilities^15^, advanced materials, and innovations in real-time data aggregation and processing during flight^16^. The increasing adoption of UAVs, particularly in autonomous transportation and entertainment, necessitates stringent reliability and safety standards^17^. To address these challenges, novel methodologies in sensor data fusion, fault detection, fault-tolerant estimation, and fault-tolerant control are being actively developed, enhancing the resilience and robustness of these autonomous systems^18–21^.

Unmanned aerial vehicles (UAVs) are highly versatile systems, but their operation is susceptible to various types of damage due to environmental, mechanical, or operational factors^22,23^. Common sources of damage include collisions, exposure to extreme weather conditions, and component failures such as motors, propellers, or battery malfunctions^24,25^. These damages can significantly impact flight stability, operational efficiency, and safety, particularly in critical applications like transportation and infrastructure inspections^26–29^. To mitigate these risks, modern UAV designs incorporate advanced fault detection systems, robust materials, and fail-safe mechanisms to enhance reliability and minimize downtime^30,31^.

Vibroacoustic analysis is a vital tool for diagnosing unmanned aerial vehicles (UAVs) by capturing and analyzing vibrations and acoustic emissions from their mechanical and structural components^32–34^. The first instance of these applications is a paper written by Rao, who used a triaxial accelerometer to observe the behavior of the entire octocopter platform and measure vibrations on each UAV arm resulting from the operation of the propulsion system, both without blade damage and with shortened rotor blades. The recorded acceleration signals for the intact drive system and damaged UAV system at two different levels of the blade fault enabled the calculation of a damage indicator based on the histogram difference and application of stochastic subspace-based fault detection algorithms, and consequently allowed for classification of the severity of the cut rotor blade^35^. Another example in the same research field is a paper written by Kulikowski, who proposed an acoustic-based FDI system for fault detection and location of the drive system working with multiple fault classes of a broken propeller^36^. Further examples include papers written by Giernacki et al., who developed a fault identification and classification system for detecting UAV propeller failures using onboard IMU sensors located on each UAV arm^37,38^. Taking into account the above-mentioned papers, it can be noticed that time-domain methods like time waveform analysis of accelerations or acoustic signals and statistical parameters (e.g., root mean square and kurtosis) help in identifying transient events and sudden changes in vibration signals^39,40^.

Frequency-domain techniques such as Fast Fourier Transform (FFT), power spectral density analysis, and envelope analysis are widely used for detecting periodic defects in components like bearings^41^ and gears^42^. Advanced methods like wavelet transforms and cepstral analysis combine time and frequency information, enabling the detection of localized defects and nonlinear behavior. By using these diagnostic techniques alongside piezoelectric sensors^43^ and machine learning algorithms, vibroacoustic analysis improves the precision of fault detection and classification^44^. The first example of this research is a paper written by Rangel-Magdaleno et al., who proposed a method for detecting an unbalanced blade on a UAV by analyzing the emitted sound. Acoustic signals were acquired during take-off and hovering, then analyzed using discrete wavelet transform (DWT) decomposition and the Fourier transform. The tests were performed using a balanced blade and an artificially unbalanced blade. The results demonstrated that the proposed methodology can distinguish between balanced and unbalanced blades^45^. These approaches contribute to predictive maintenance strategies, minimizing operational downtime and extending the lifespan of UAVs^46^.

Electric motor failures in drones can severely impact performance, safety, and reliability, often leading to costly downtime or catastrophic failure. Common issues include winding insulation breakdown due to thermal stress, bearing wear from dust, moisture, or poor lubrication, rotor imbalance caused by defects or debris, and demagnetization of permanent magnets from high temperatures or external magnetic fields^47–49^. These failures lead to short circuits, increased friction, vibrations, reduced efficiency, and impaired maneuverability, while electrical faults such as phase loss can cause irreparable damage. Vibrations from these issues can affect onboard sensors, further degrading performance, while overheating due to poor ventilation or overloading shortens motor lifespan. Analyzing the effects of failure is crucial to understanding its root causes, predicting potential cascading issues, and developing effective mitigation strategies to ensure system reliability. Early detection using tools like piezoelectric sensors, thermal imaging, or machine learning, alongside predictive maintenance and optimized motor design, is essential to enhance reliability and prevent critical failures.

Despite advancements in drone technology, the impact of electric motor failures on the structural integrity of drones remains an underexplored area^50–52^. Existing studies primarily focus on motor performance and fault detection, often neglecting how these failures propagate through the drone’s frame and affect its structural health. Vibrations caused by issues like rotor imbalance, bearing wear, or demagnetization can resonate with the drone’s natural frequencies, leading to fatigue and potential micro-cracks in critical components. The lack of precise diagnostic tools to quantify this structural impact further compounds the problem. Piezoelectric sensors offer a promising solution for capturing high-resolution vibration data, but their integration into drones for structural diagnostics remains underutilized. Combining such sensor data with machine learning methods could enable predictive models to link motor failures with structural damage patterns, providing a deeper understanding of this interaction. Addressing this research gap could improve drone design, enhance fault tolerance, and extend operational lifespan.

Recognizing the ongoing need for improving drone reliability, this study examines how damage to one octocopter motor affects the structure. To achieve this, a windowing method was employed to calculate statistical indicators in time and frequency domains, which were then applied to machine learning models for automatic damage detection and prediction. Tests were conducted at different PWM duty cycles in Ground and Fly Modes. The models showed the highest accuracy in detecting damage on the affected drone arm, while the lowest accuracy appeared on opposite arms. This approach allows us to assess the impact of damage on the entire drone structure and understand how the damage propagates to other motors of the octocopter. Such analysis is essential for drone applications in critical fields like search-and-rescue, defense, and industrial inspections, where early fault detection can prevent mission failures and enhance operational safety.

The key novel contributions of this study are as follows: (1) We present a UAV diagnostic approach that investigates not only the location of motor damage but also its structural propagation across the UAV frame; (2) we implement piezoelectric sensors on all arms of an octocopter and analyze the spatial distribution of vibrations induced by controlled motor degradation; (3) we apply statistical features extracted from both time and frequency domains in a short-time windowing scheme to enhance classification sensitivity; and (4) we compare multiple machine learning models to evaluate damage detectability on each UAV arm, providing a detailed accuracy map and demonstrating that frequency-domain features are more effective on distant arms. This comprehensive approach contributes to the development of predictive UAV health monitoring systems with greater fault localization and severity resolution.

The entire process is described in the following sections. Section 2 outlines the experimental setup and measurement system used to capture high-resolution vibration data, while Section 3 details the signal processing and feature extraction process for identifying failure patterns. In Section 4, machine learning models analyze these patterns to explore the correlation between motor faults and structural damage, providing valuable insights for improving drone reliability and fault tolerance. In Section 5, the discussion of results is presented, while Section 6 summarizes the research.

## Proposed approach and methodology

**Fig. 1:**
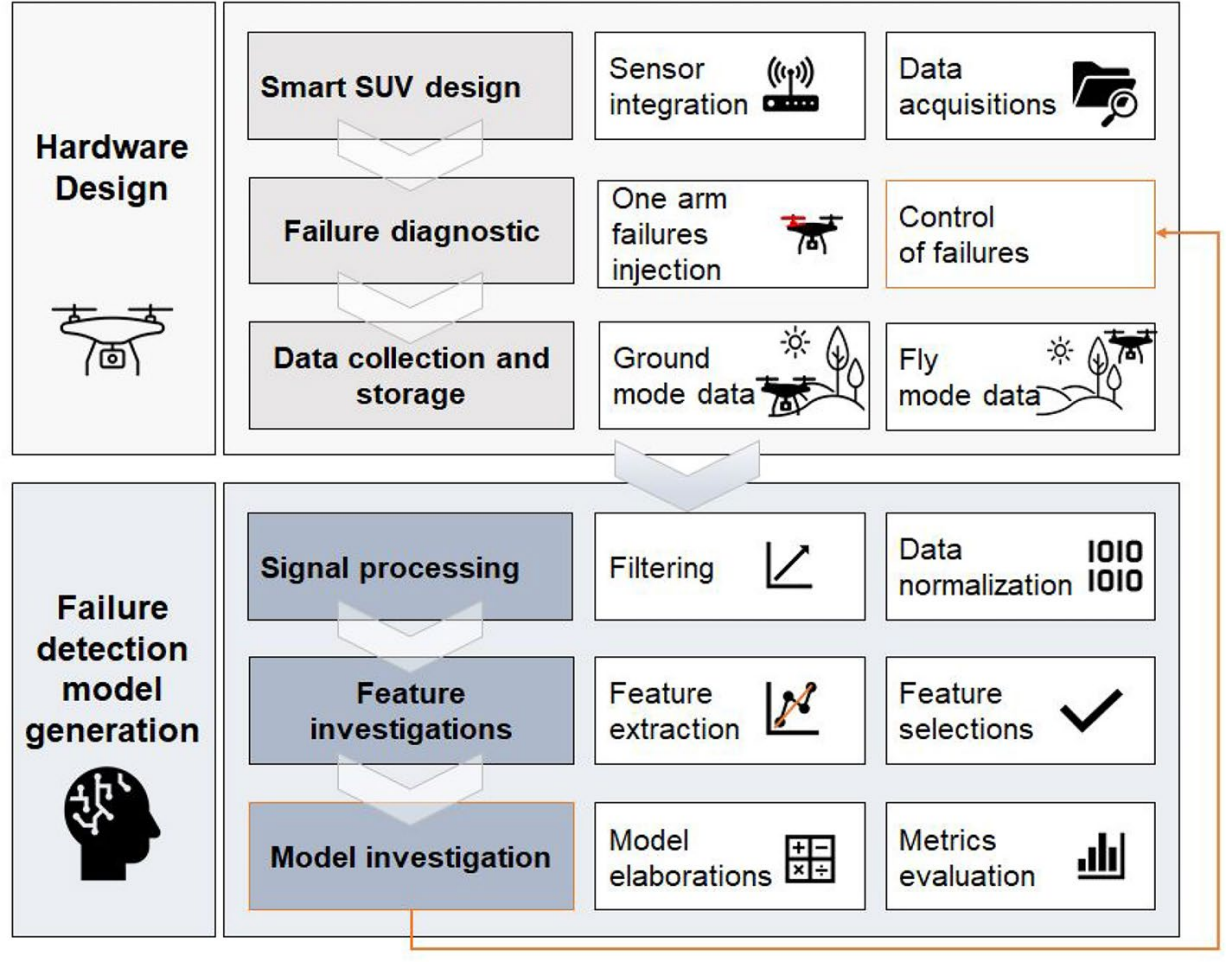
Proposed methodology for UAV electric motor failure detection.

The proposed approach focuses on diagnosing the failure of a UAV’s drive system using piezoelectric sensors and machine learning techniques. The proposed methodology, shown in Fig.1, is based on an experimental investigation with an appropriate hardware design for the UAV system, as well as the analysis of the collected data and the generation of an optimal machine learning model that can later help control the UAV in the event of damage. In the case of the experiment, ground mode and fly mode tests were conducted.

## Hardware design and data collection

**Fig. 2:**
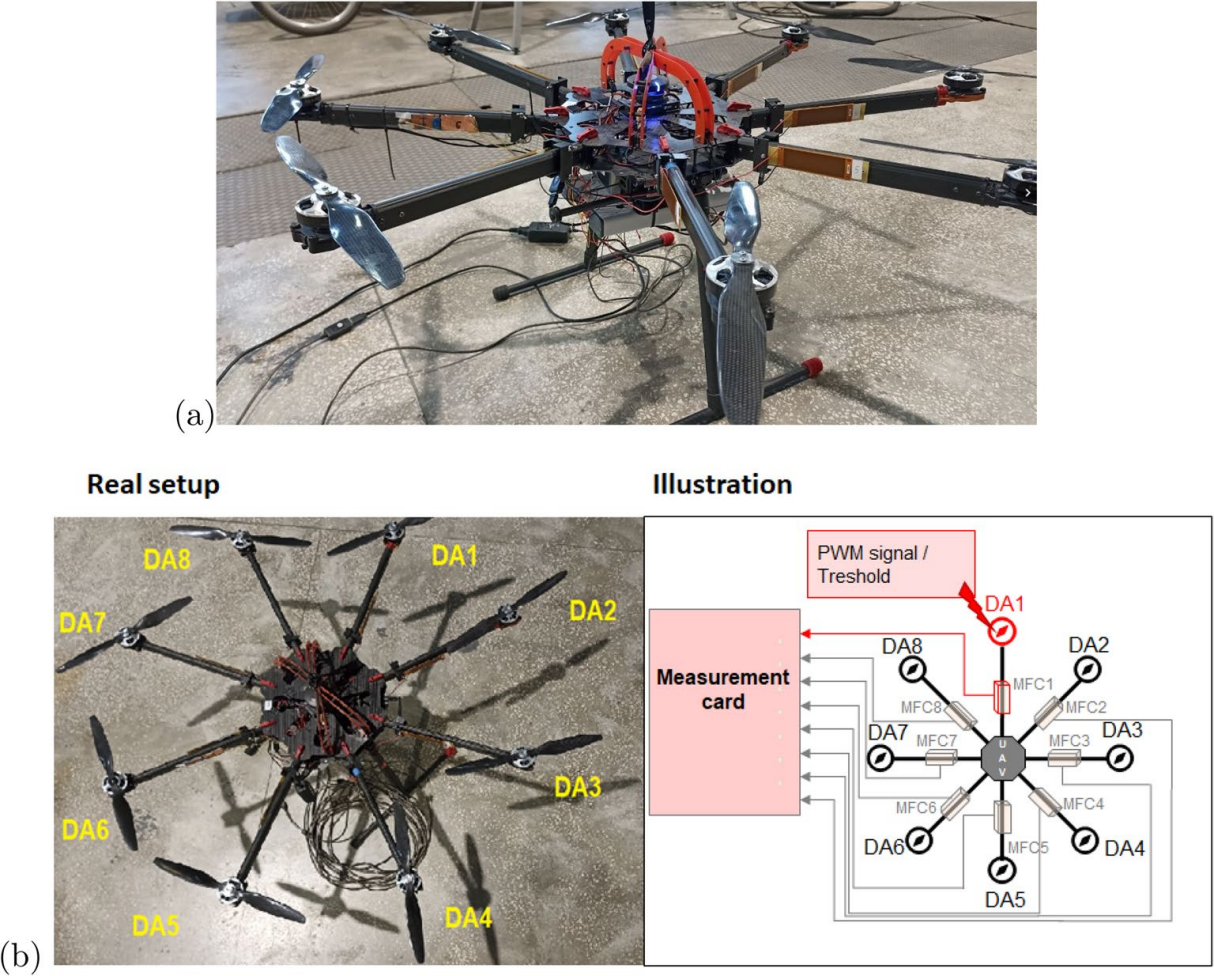
The view of VTOL UAV used in the experimental tests with the marked drone’s arm (DA) (**a**) isometric view, (**b**) the top view of the UAV structure consisting of drone, communication system and data acquisition system.

In the experimental setup, failure diagnostics of the VTOL drive unit system (see Fig.2) were conducted using commercial macro-fiber composite sensors. The drive system, developed by researchers at Bialystok University of Technology (Poland), is mounted on a UAV platform with a carbon composite center plate and aluminum arms (Fig. 2a). Powered by a 6-cell LiPo battery (22.2V), the propulsion system consists of eight 15 $\times$ 5.5 propellers directly attached to Tarot 5008 BLDC motors. An autopilot Pixhawk controls flight stability and maneuverability, communicating with ESCs to regulate motor speed. Additionally, in Fig. 2b, shows the scheme depicting the connection between the multirotor and the data acquisition system consists of an 8-channel board of type NI USB-6351, along with a way threshold/damage to the propulsion system is presented. Also, the list of the most important hardware used in the experiment is presented in Table 1. The following subsections detail the measurement system and piezoelectric output analysis.

**Table 1:** Hardware and Software used in the experiment.

Component	Type
Electric motor	Tarot 5008 BLDC Motor
Electronic speed controller	Hobbywing 40A
Autopilot	Pixhawk Cube
Piezoelectric sensor	M8528P2 (Smart Materials - Dresden)
DAQ Board 1	NI USB-6351
DAQ Board 2	Analog Discovery 2
DAQ Software	NI FlexLogger (Board 1) Waveform (Board 2)

### Measurement system

**Fig. 3:**
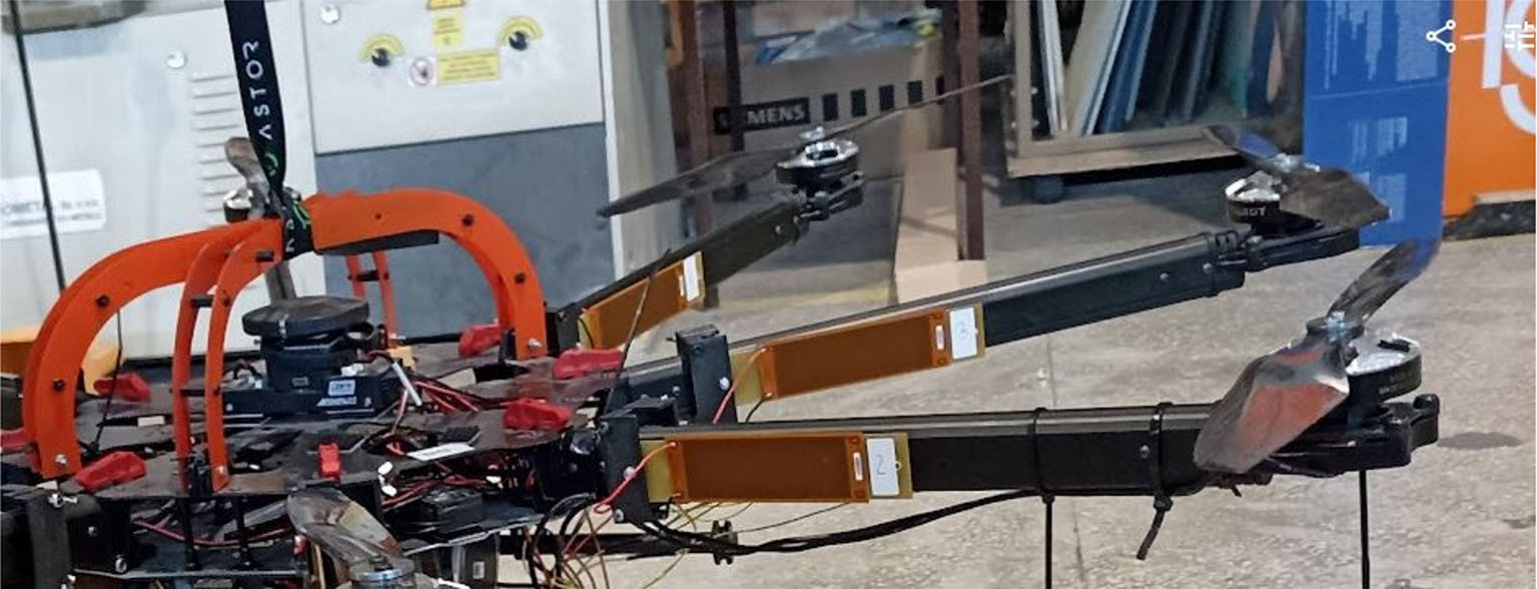
Illustration of piezo composite sensors located on the side plane of the UAV arm.

**Fig. 4:**
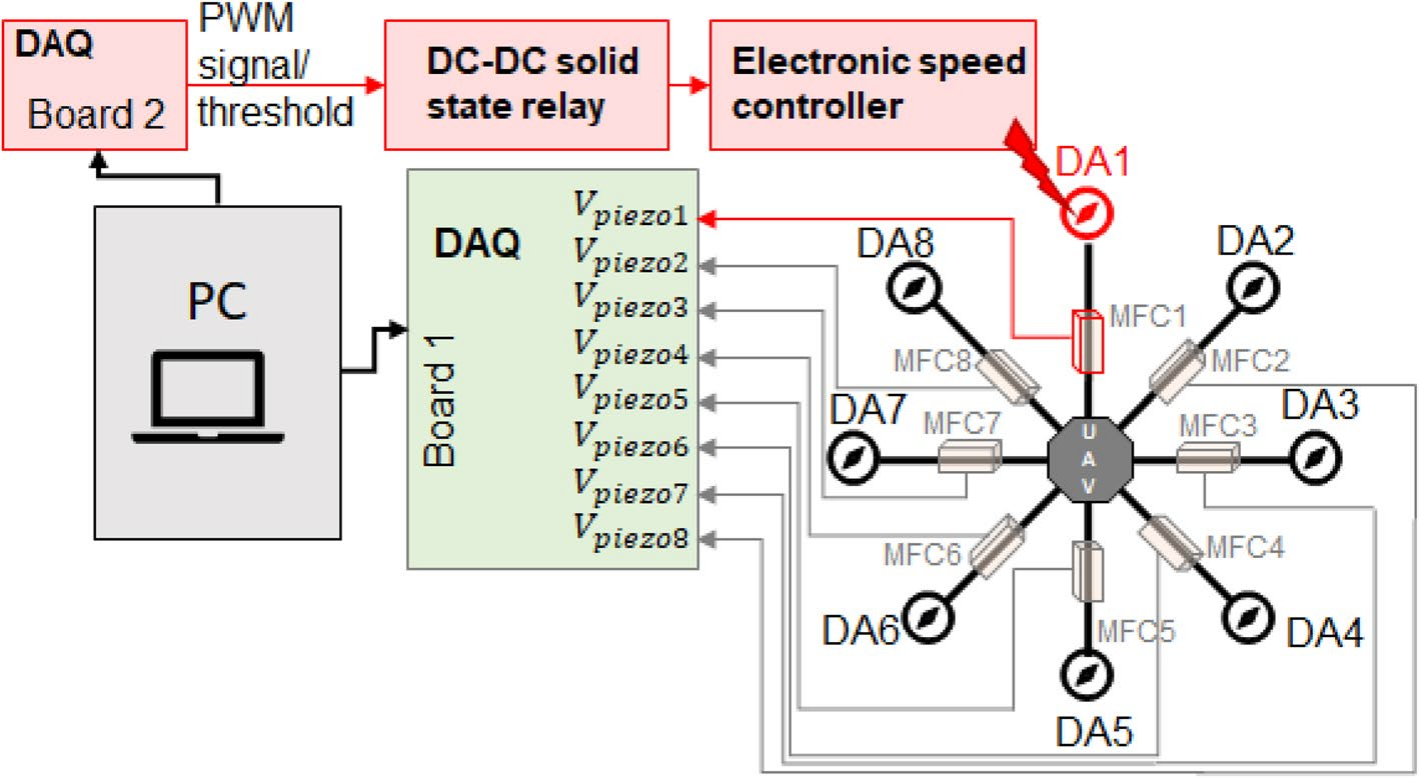
Diagram of the proposed measurement system.

To study the impact of damage level on the drone’s dynamics, the UAV platform was equipped with the proposed measurement system , which includes eight MFC piezo-sensors of type 8528 P2 located on each arm of the octocopter, as well as two additional measurement cards and the DC-DC Solid State Relay placed below the autopilot Pixhawk. As shown in Fig. 3, the aforementioned MFC sensors were mainly used to measure the arms’ vibrations coming from the drive system working in different states under different levels of damage based on different PWM levels. The placement of these sensors on the side plane of each arm was the result of numerical analysis of the FEM model of this arm and experimental tests, which results were described in detail in the following papers^47,48^. The outcome of the previous research was the identification of the most optimal mounting position of the sensor on the drone’s arm and the accuracy of the recurrence analysis in the recognition of small amplitude vibrations with transient characteristics coming from the damage, which is why the sensors are mounted on the lateral plane of the arm. Moreover, the proposed measurement system depicted in Fig. 4 was also retrofitted with second separate 2-channel measurement card of type Analog Discovery 2 developed by the Digilent company that together with the Solid State Relay was used to switch on/off one from three phases applied to the brushless motor located on chosen UAV arm. In this case, the application of the SSR was necessary to obtain the appropriate switching frequency and pass a relatively high amplitude electrical current, even up to 30A, to the drive system through the electronic speed controller (ESC).

**Fig. 5:**
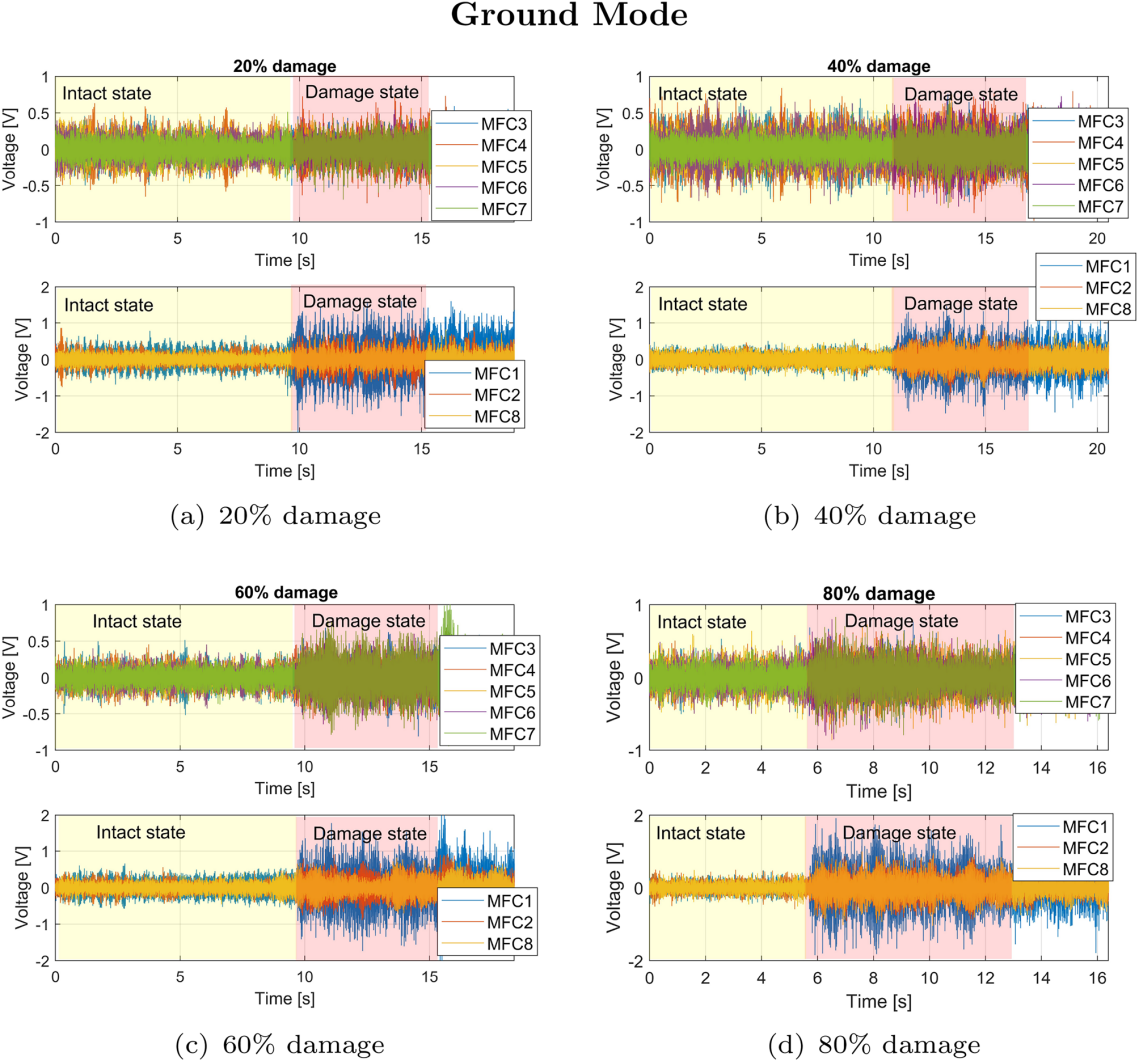
Voltage generated by macro-fiber composites located on each arms during Ground Mode test by various level of damage of the electrical motor *M1* equal (**a**) 20%, (**b**) 40%, (**c**) 60%, (**d**) 80%.

**Fig. 6:**
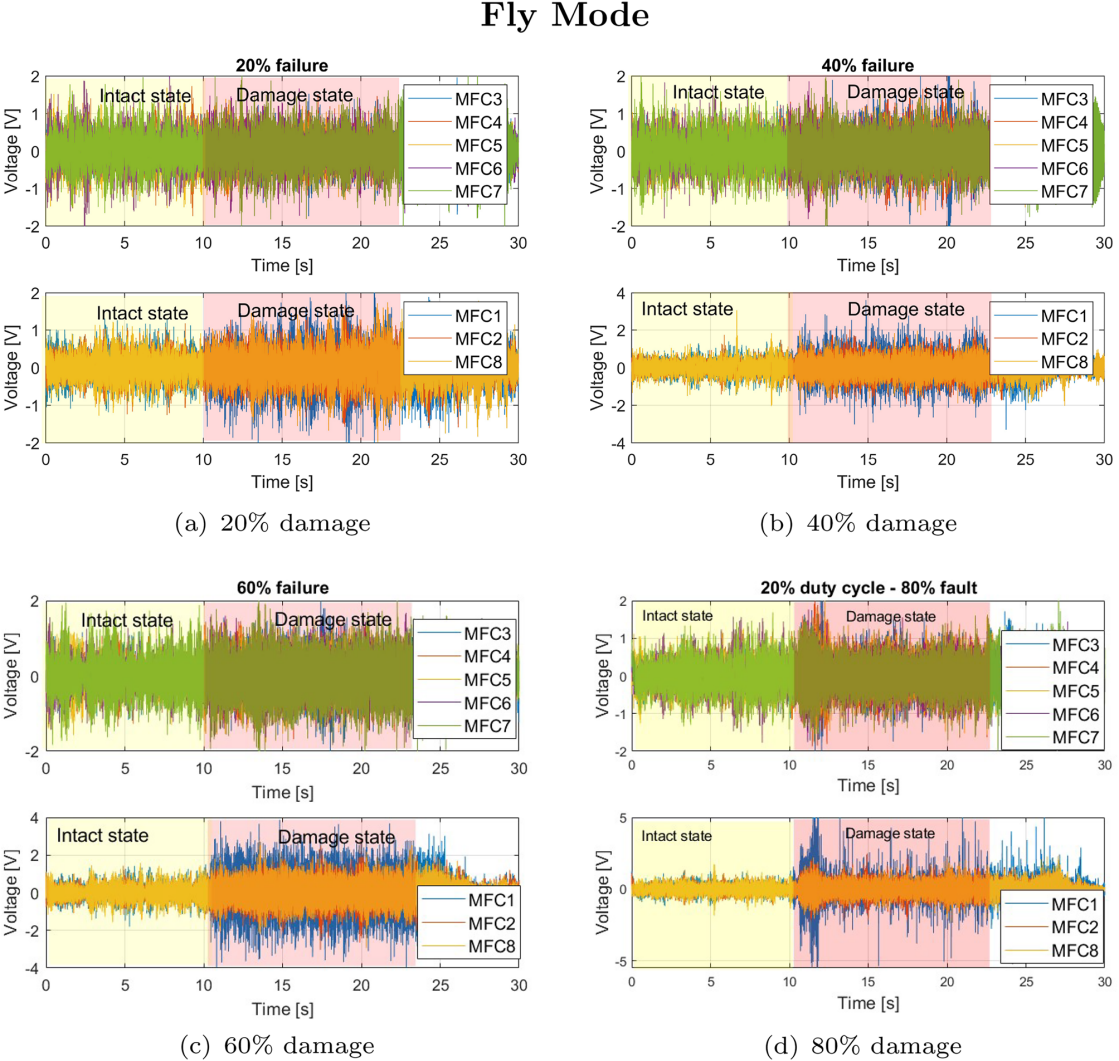
Voltage generated by macro-fiber composites located on each arms during Fly Mode test by various level of damage of the electrical motor *M1* equal (**a**) 20%, (**b**) 40%, (**c**) 60%, (**d**) 80%.

The tests were conducted in two configurations, i.e. when the UAV was seated (Ground Mode) and in flight (Fly Mode). Both experimental indoor tests on failure detection of the UAV drive system working under low (throttle set to 15%) load and full load (throttle set to 60%) were carried out for the grounded and flying UAV platform, respectively , by four different levels of the electrical motor damage. Video recordings of these tests are accessible via the following links: Ground Mode and Fly Mode. In both cases, one of the eight of the BLDC motor (M1) located on the drone arm (DA1) was temporarily damaged by applying to its two of six coils the PWM control signal with the progressively reduced duty cycles equal 80%, 60%, 40%, and 20%, (the detailed graphics showing control of PWM level is presented in the previous article^53^) respectively. As a result, it led to an inverse effect in the form of increasing motor damage and, consequently, loss of lift force generated by this drive system. Considering this fact, the voltages generated by all piezo sensors located on each arm DA1-DA8 for the grounded UAV platform and the flying UAV platform for each time were measured for each test by the 8-channel measurement card. As a result, the recorded voltage output signals from two other tests are shown in Fig. 5 and Fig. 6, respectively. All voltage time-series taken for analysis were collected in the same way, first 10 seconds of the flight for the stable position, while for the next 10 seconds for the multirotor with the initiated damage. For the binary classification, an equal number of data points from intact and damaged mode by each load case was taken for further analysis. The analysis of time-series in both the time and frequency domains provides a comprehensive understanding of system behavior, capturing transient and steady-state characteristics while enabling the detection of patterns, anomalies, and underlying frequencies critical for fault diagnostics and signal processing. The overview of two methodologies will be presented in the following subsections.

### Time domain overview

The analysis of the piezoelectric outputs generated by all MFC sensors for the grounded UAV platform and the power system working under low load (Ground Mode) indicated that the placement of sensors plays a crucial role ensuring proper failure detection of the UAS (Unmanned Aerial System). Observing the diagrams presented in Fig. 5a–c determined for the low level of damage (20%, 40% and 60%) shows that only three sensors (MFC1, MFC2, MFC8) located close to the damaged electrical motor generated higher amplitude of the voltage at the moment the failure appeared in the drive unit system as well as a few seconds later when the chosen motor still operated in the damage state. In the case of other sensors (from MFC3 to MFC7), the effect of electrical motor failure was hardly measurable due to the far-away arrangement of these sensors relative to the location of the damaged electrical motor. Another effect can be shown in Fig. 5d, where all piezo composite sensors located on the UAV platform detected the damage state of the chosen motor. The main reason for this behavior was the higher vibration of the UAV arm with the BLDC motor failure hl, as well as the entire multirotor platform, due mainly to the jerking of the electrical motor chosen.

To verify these results, the dynamic indoor tests were also carried out for the drive unit system working under full load on the same flying multi-rotor (Fly Mode). To do this, the defined duty cycle of the PWM control in the limited range 20–80% was applied again to the electrical motor *M1* with the help of the Solid Switch Relay. The recorded signals again showed the highest increase in the voltage amplitude generated by the MFC1 sensor as well as the relatively higher voltages generated by two other piezosensors MFC2 and MFC8, especially at the time of appearance of failure in the drive unit system due to 20%, 40% and 60% damage of the chosen electric motor (see Fig.6a–c). Similarly to the previous test, another behavior can be observed for a higher level of damage equal to 80% (see Fig.6d), where a moment of appearance of the failure in the electrical motor *M1* was detected by all piezo sensors located on the platform. As a result, the amplitude of voltage generated by the piezo MFC1 was five times higher compared to the amplitude of voltage generated by this sensor for the undamaged motor. In contrast, in the case of other piezo sensors, these increases were higher than twice.

### Frequency domain overview

For the frequency domain analysis of the time series, a Fast Fourier Transform (FFT) was performed for two states of the electrical motor *M1* (undamaged and damaged), separately. Each time, 9-second voltage signals from onboard piezo composite sensors MFC were analyzed separately with no overlap ratio using a sampling frequency of 1kHz and an FFT resolution of 0.111Hz. In addition, all power spectra were determined for the drive unit system operating in the grounded UAV (Ground Mode) and the flying platform (Fly Mode) by varying the duty cycle values of the PWM control signal, from 20% to 80% in 20% increments.

**Fig. 7:**
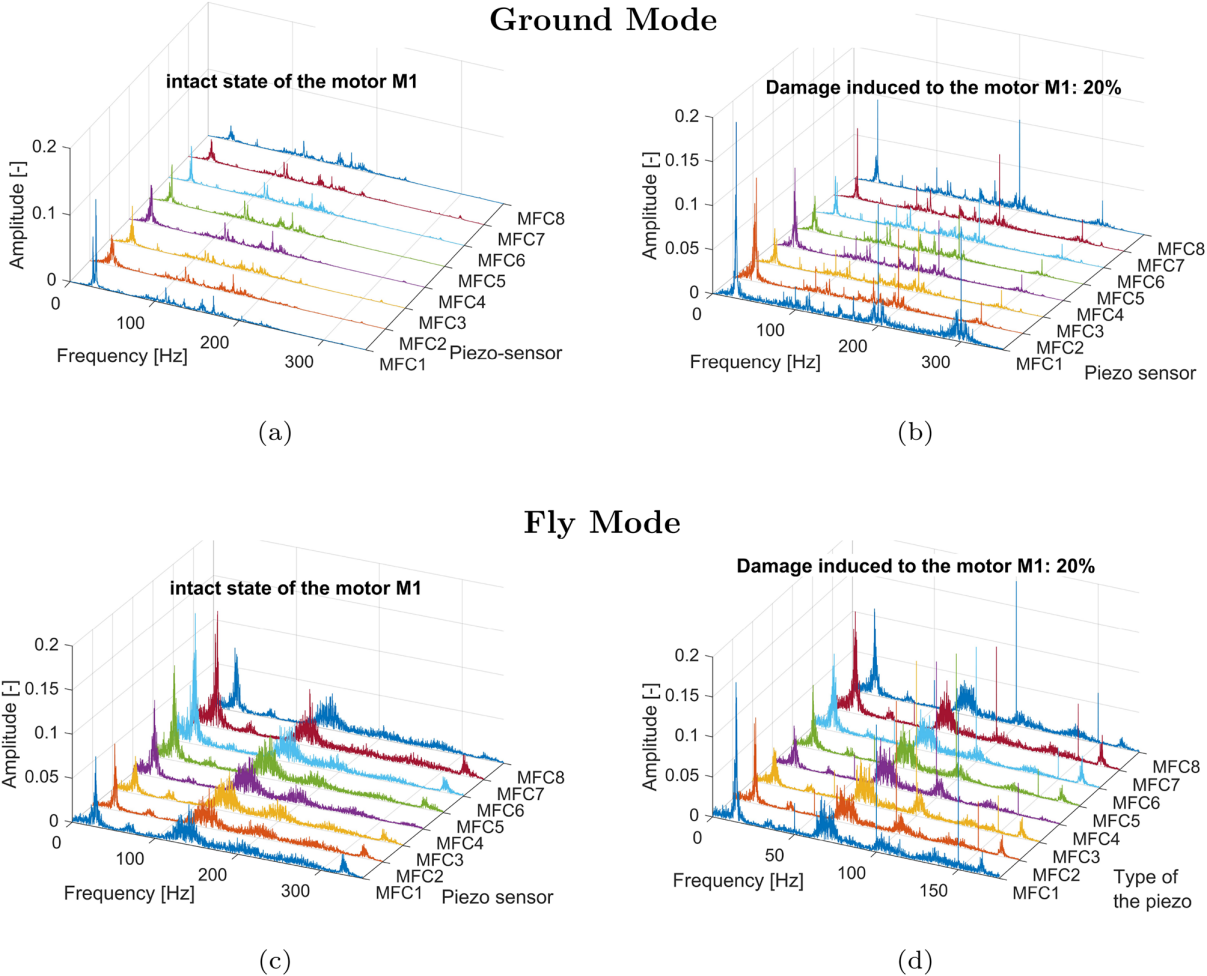
Comparison of the power spectrum of the voltage signals generated by the piezo sensors MFC1-MFC8 for (**a**) the intact state of the motor *M1* for the grounded UAV (**b**) failure state of the motor *M1* by 80% damage for the grounded UAV (**c**) the intact state of the motor *M1* for the flying UAV (**d**) failure state of the motor *M1* by 80% damage for the flying UAV.

**Fig. 8:**
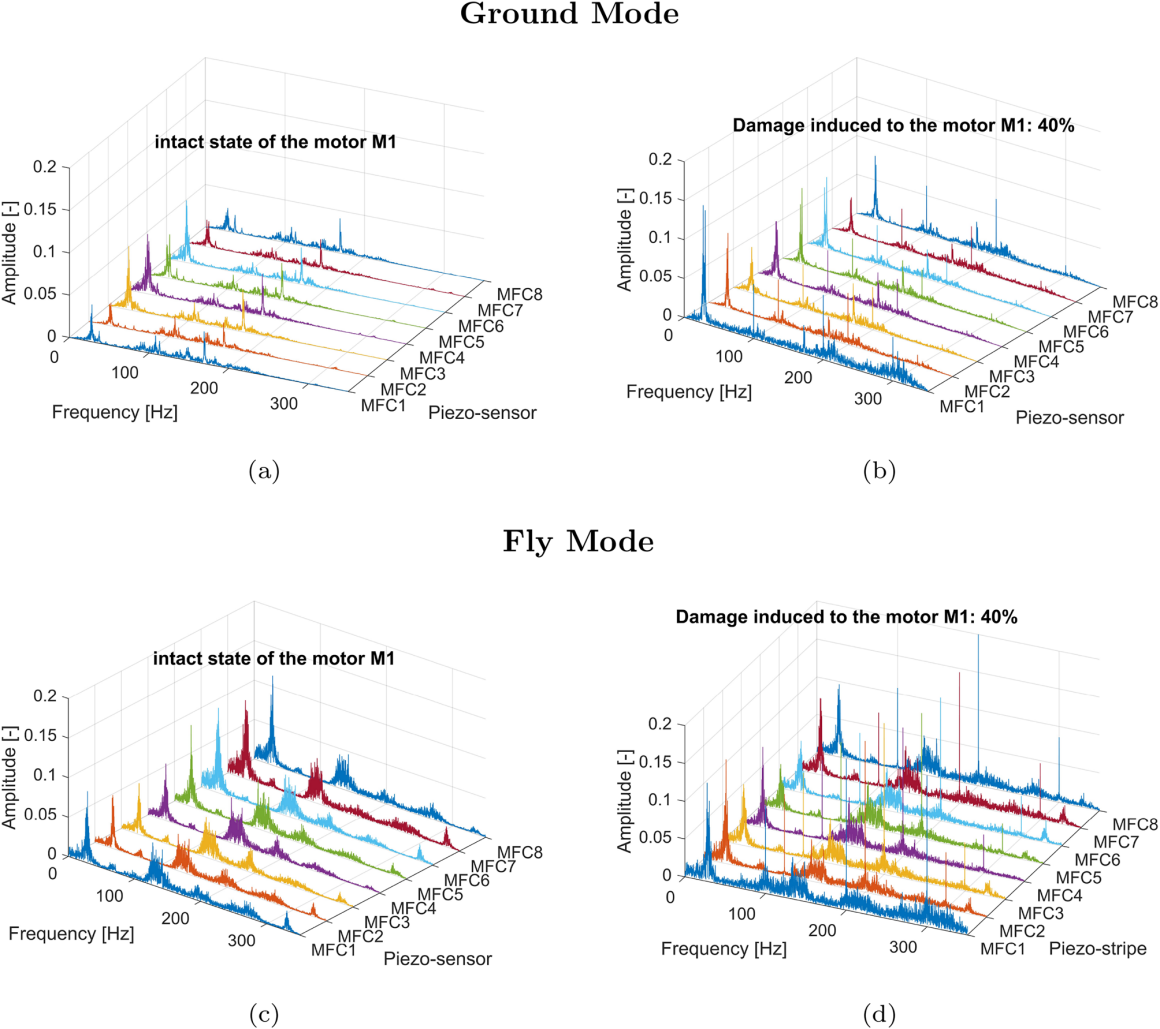
Comparison of the power spectrum of the voltage signals generated by the piezo sensors MFC1-MFC8 for (**a**) the intact state of the motor *M1* for the grounded UAV (**b**) failure state of the motor*M1* by 40% damage for the grounded UAV (**c**) the intact state of the motor *M1* for the flying UAV (**d**) failure state of the motor*M1* by 40% damage for the flying UAV.

**Fig. 9:**
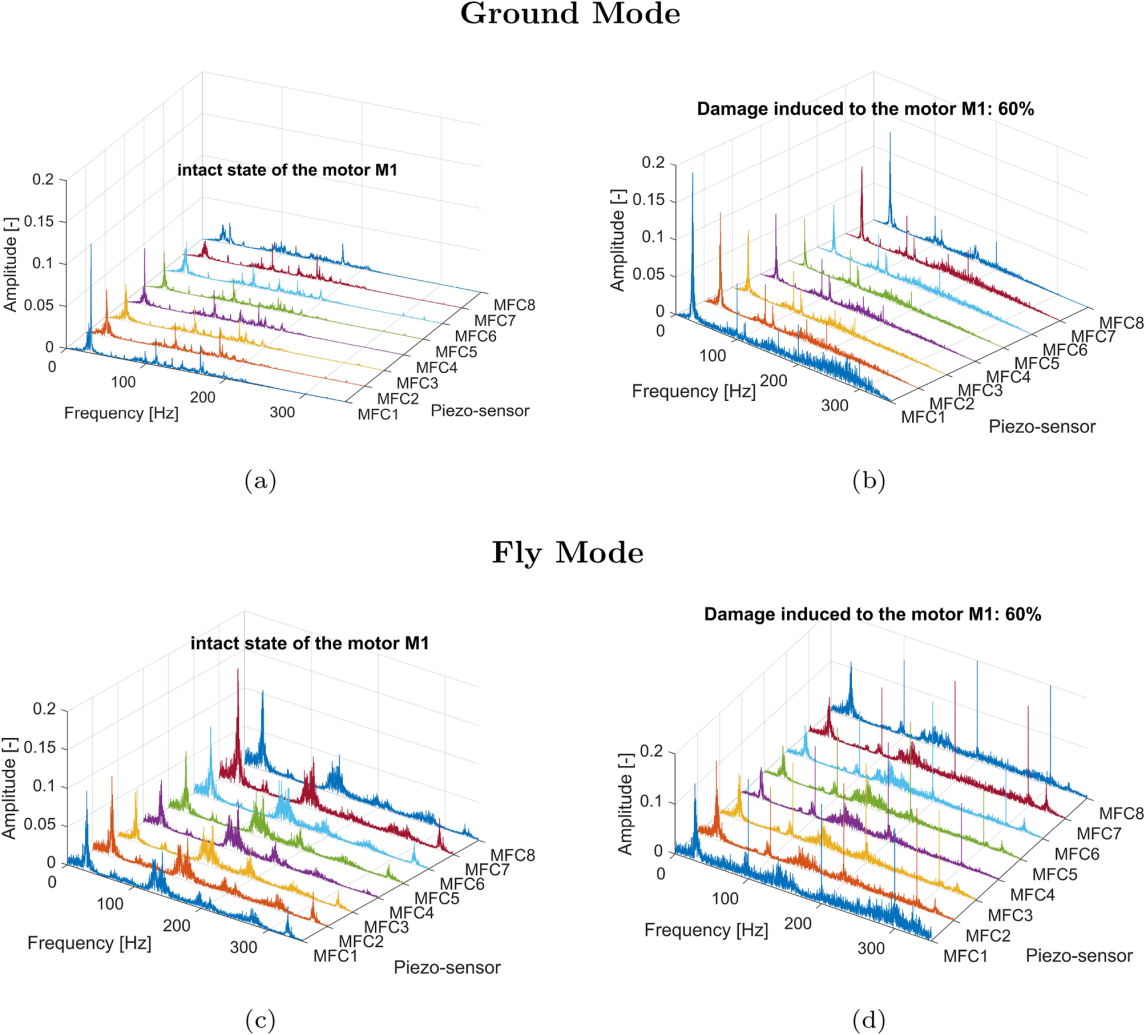
Comparison of the power spectrum of the voltage signals generated by the piezo sensors MFC1-MFC8 for (**a**) the intact state of the motor *M1* for the grounded UAV (**b**) failure state of the motor *M1* by 60% damage for the grounded UAV (**c**) the intact state of the motor *M1* for the flying UAV (**d**) failure state of the motor *M1* by 60% damage for the flying UAV.

**Fig. 10:**
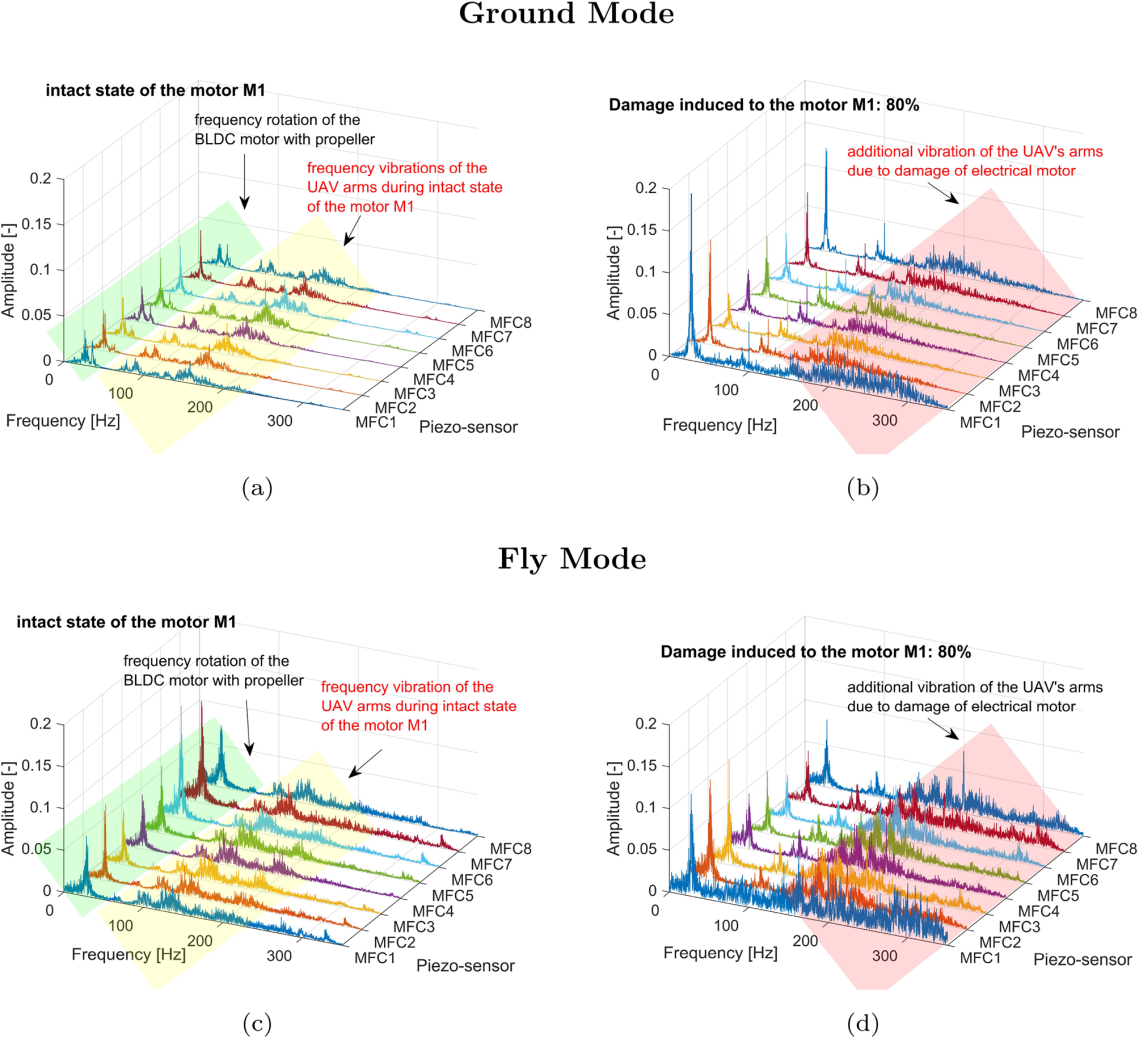
Comparison of the power spectrum of the voltage signals generated by the piezo sensors MFC1-MFC8 for (**a**) the intact state of the motor *M1* for the grounded UAV (**b**) failure state of the motor *M1* by 20% damage for the grounded UAV (**c**) the intact state of the motor *M1* for the flying UAV (**d**) failure state of the motor *M1* by 20% damage for the flying UAV.

The conducted analysis of the results presented in Fig.7a and c indicated that the frequency range of the undamaged drive unit system working under low and high loads is limited up to 200 Hz. In both cases, the rotational velocity of the electrical motor and the frequency vibration of particular arms of the UAV were equally detected by all eight piezo-sensors located on this UAV platform. Other behaviors can be observed in Fig. 7b and d for the damaged state of the electrical motor where a rapid change in duty cycle of the PWM control signal from 100% to 20% corresponding 80% damage of this motor led to the appearance of additional low amplitudes vibrations on the UAV platform in the frequency range over 200 Hz mainly measured by the piezo sensor MFC1 and the piezo sensor MFC8, respectively. In addition, the sudden change of pulse width in the PWM control signal affected an increase of the amplitude peaks relating to the rotation speed of the motor as well as the amplitude vibrations of the UAV platform, which again were mainly measured by piezo sensors MFC1 and MFC8. Similar behavior can be observed also during the analysis of the power spectrum shown in Figs. 8 and 9 where significant jerking of the electrical motor *M1* by both 40% and 60% damage of this motor placed on the flying UAV platform again led to appear additional low-amplitude vibrations of the UAV arms over 200Hz. However, completely different behavior can be observed for 20% damage introduced to the drive unit system of the UAV, where a limitation of the PWM duty cycle from 100% to 80% did not significantly affect the shape of the power spectrum presented in Fig. 10. As a result, all four power spectra generated for the intact (Fig. 10a and c) and damaged state (Fig. 10b and d) of the electrical motor *M1* allowed only to indicate frequencies related only to the rotation speed of the electrical motor and vibrations of the particular eight arms of the UAV platform.

**Fig. 11:**
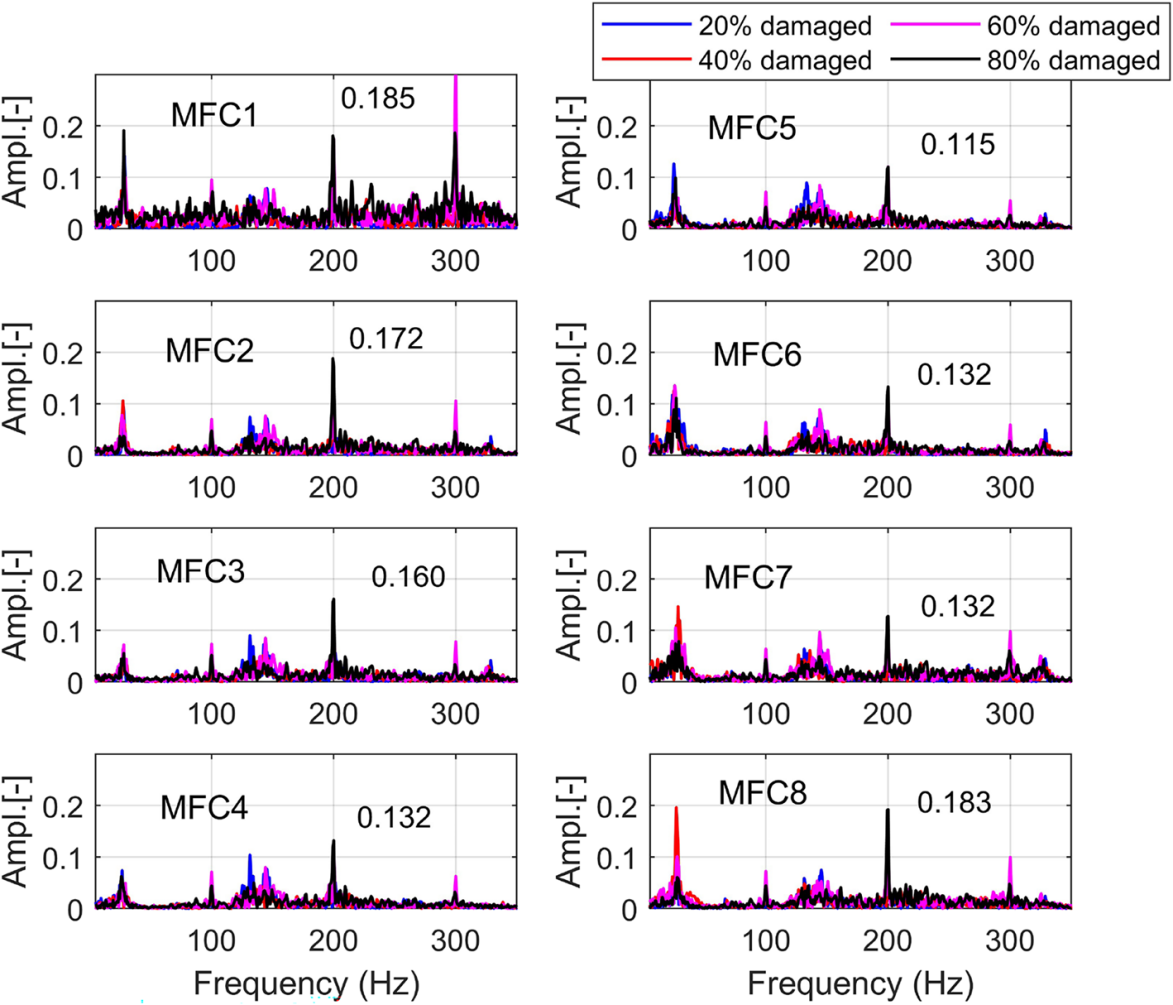
Comparison of the power spectrum of voltage signals generated by all onboard piezo stripe sensors MFC1-MFC8 by 20%, 40%, 60%, 80% damaged induced to the motor M1.

Moreover, analysis of the power spectrum presented in Fig. 11 indicated that changing the duty cycle of the PWM signal applied to the motor M1, especially at higher damage levels in the range 40–80%, leads to the appearance of additional high peaks in the frequency equal 200Hz and 300Hz which is due to characteristic motor phase asymmetric or winding failure. The aforementioned peaks were measured by all onboard piezo stripe sensors, and as expected, the highest amplitudes were obtained by sensors MFC1, MFC2 and MFC8 (0.185; 0.172; 0.182) locating the closest to the damage motor, while the lowest amplitude (0.115)—by the sensor MFC5 located far away from the motor working with damage (opposite arm DA5 of the UAV). As a result, considering the obtained results as well as to automatically recognize the damage-level and related signals’ characteristics related to it, the short-time series window analysis is recommended. To do it, firstly, the statistical features based on time and frequency domains should be calculated, which are described in detail in the following Section.

## Feature extraction process

**Fig. 12:**
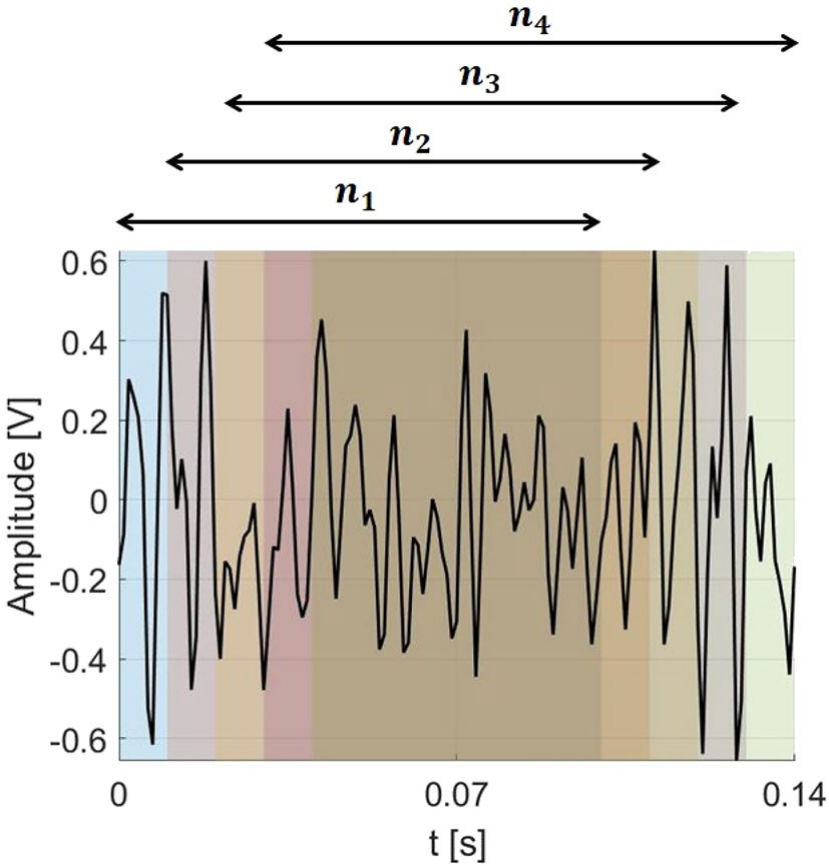
Example of an output voltage signal. The figure highlights fixed-width windows, each consisting of 100 measurement points. Consecutive windows overlap, i.e., $n_{1}$ covers data points 1-101, $n_{2}$ covers data points 11-111, $n_{3}$ covers data points 21-111, $n_{4}$ covers data points 31-131, and so on, in which the indicators are calculated.

**Fig. 13:**
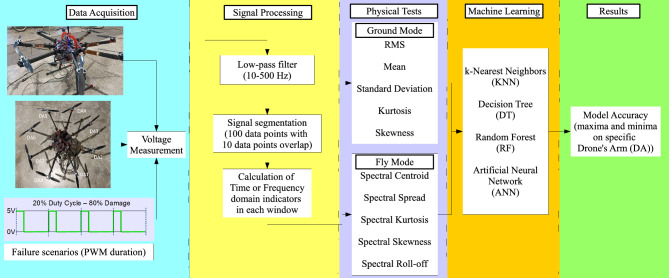
Flowchart representing the entire process, starting from the conducted experiment, followed by signal processing, indicator calculations, machine learning model creation, and data analysis.

Feature extraction methods were employed to obtain useful indicators corresponding to the system dynamics based on raw voltage signals recorded by all piezo composite sensors located on each arm of the drone. These features were determined in the time, frequency, and time-frequency domains. Depending on the chosen domain, they can identify different properties of the signal. The easiest to extract are time-domain features based on moments, such as mean, variance, skewness, and RMS^55,56^. These features can also be transformed into the frequency domain and calculated based on the power spectral density. In this domain (frequency domain), they correspond to frequency components that reflect the state of the drive system. Finally, five time-domain features like RMS, mean ($\mu$), standard deviation ($\sigma$), kurtosis ($\gamma _1$), and skewness ($\gamma _2$) given by Eqs. (1)–(5) as well as five frequency-domain features: spectral centroid ($\mu _{spe}$), spectral spread ($\sigma _{spe}$), spectral kurtosis ($S_{Kurt}$), spectral skewness ($S_{Skew}$) and spectral roll-off ($S_{Roll-Off}$) given by Eqs. (6)–(8) were calculated. In both cases, all indicators according to Fig. 12 were determined in overlapping windows of 0.1 seconds with an overlap of 0.01 seconds. The main advantages of window analysis in short time series include enhanced robustness by analyzing overlapping segments^57,58^, reducing noise effects, and capturing temporal dynamics related to the time-limited start of the defect in one of the electric motors. Moreover, it also facilitates feature extraction and anomaly detection, making it valuable for predictive modeling and diagnostics, which will be conducted in the next Section referring to the Machine Learning Analysis. Before the feature calculation in short-time series windows, the signals were filtered with the band-pass filter in the frequency range 10-500Hz, which is presented in Fig. 13.

### Time-domain features

Time-domain features such as RMS, mean, standard deviation, skewness and kurtosis given by Eqs. (1)–(5) provide valuable insights into signal characteristics^59–63^. All of them are useful tools in the identification of anomalies occurring in the voltage signals. RMS reflects the overall energy of the signal, helping to detect variations due to defects, while the mean captures general shifts in vibration level^59^. Standard deviation quantifies signal dispersion, indicating instability or increased variability caused by various levels of damage^60^, while kurtosis and skewness highlight deviations from a normal distribution^61,62^, where high level of kurtosis refers to the impulsive events, and skewness detects asymmetry in signal fluctuations, both useful for identifying localized defects. The above-mentioned features enable a detailed assessment of the drone’s condition under different levels of damage. Their application in short-time series analysis improves diagnostic accuracy, making them essential tools for real-time condition monitoring.

1 $$\begin{aligned} \text {RMS}= & \sqrt{\frac{1}{N} \sum _{i=1}^{N} x_i^2} \end{aligned}$$

2 $$\begin{aligned} \mu= & \frac{1}{N} \sum _{i=1}^{N} x_i \end{aligned}$$

3 $$\begin{aligned} \sigma= & \sqrt{\frac{1}{N}\sum _{i=1}^{N} (x_i^2-\mu ^2)} \end{aligned}$$

4 $$\begin{aligned} \gamma _1= & \frac{N}{(N-1)(N-2)} \sum _{i=1}^{N} \left( \frac{x_i - \mu }{\sigma } \right) ^3 \end{aligned}$$

5 $$\begin{aligned} \gamma _2= & \frac{N(N+1)}{(N-1)(N-2)(N-3)} \sum _{i=1}^{N} \left( \frac{x_i - \mu }{\sigma } \right) ^4 - \frac{3(N-1)^2}{(N-2)(N-3)} \end{aligned}$$

where: *N* is the number of samples in the overlapping window, $\mu$ is the mean, $\sigma$ is the variance while $x_i$ is the *i*-th value of the recorded voltage signal in the overlapping window.

### Frequency-domain features

Contrary to time-domain features, frequency-domain features provide crucial insights into the spectral characteristics of the signals, making them valuable tools for anomaly detection. These features help assess variations due to defects by analyzing how signal energy is distributed across different frequencies within the signal^63^. The first of the applied features is the spectral centroid, representing the center of mass of the power spectrum, indicating the dominant frequency component. A shift in the spectral centroid may suggest wear or damage in mechanical components^64^. The next is the spectral spread quantifying the dispersion of spectral energy around the centroid, helping to detect instability and increased variability in signal frequency distribution^65^. Spectral kurtosis is responsible for measuring the peakedness of the spectral distribution, where high values indicate impulsive spectral phenomena that are often related to sudden faults or localized defects^66^. Spectral skewness is strictly associated with the above-mentioned features and it describes the asymmetry of the spectral distribution, revealing changes in signal fluctuations and the presence of frequency-domain anomalies^67^. The last of the used features is the spectral roll-off defining the frequency below which a given percentage (usually 85% or 95%) of the spectral energy is concentrated. A shift in roll-off frequency can indicate structural changes in the propeller due to progressive damage^68^. The above-described features enable a detailed evaluation of a drone’s condition at various damage levels. Below, the mathematical formulas of the spectral for the mentioned features are expressed as^63–68^:

6 $$\begin{aligned} {S_{Skew}}= & \frac{\sum _{k=b_1}^{b_2} (f_k - \mu _{spe})^3 X_k}{\left( \sum _{k=b_1}^{b_2} X_k \right) \cdot \sigma _{spe}^3} \end{aligned}$$

7 $$\begin{aligned} {S_{Kurt}}= & \frac{\sum _{k=b_1}^{b_2} (f_k - \mu _{spe})^4 X_k}{\left( \sum _{k=b_1}^{b_2} X_k \right) \cdot \sigma _{spe}^4} \end{aligned}$$

8 $$\begin{aligned} {S_{Roll-Off}}= & f_r, \quad \text {where} \sum _{k=0}^{r} X_k =0.85 \cdot \sum _{k=0}^{N-1} X_k \end{aligned}$$

where $\mu _{spe}$ - the spectral centroid as well as $\sigma _{spe} - \text {the spectral spread}$ are in the following forms^63^:

$\mu _{spe} = \frac{\sum _{k=b_1}^{b_2} f_k X_k}{\sum _{k=b_1}^{b_2} X_k}, \sigma _{spe} = \sqrt{\frac{\sum _{k=b_1}^{b_2} (f_k - \mu _{spe})^2 X_k}{\sum _{k=b_1}^{b_2} X_k}},$ $f_k$ is a frequency corresponding to the *k* bin, $b_1$ and $b_2$ are the band limits in bins.

Time-domain features are more intuitive and easier to compute, making them useful in many applications, including monitoring the condition of the propulsion system of an unmanned measurement vehicle, where capturing significant changes over time or limited computational resources are crucial. On the other hand, frequency-domain features offer deeper insights into the signal structure, which can be critical in more advanced analyses.

The analysis of damage identification based on its severity (from 20% to 80%) and its propagation to the remaining arms was conducted based on the effectiveness of machine learning models in the binary classification problem. In all cases, features were generated using sliding windows based on signals corresponding to two states of the drive system on the MFC1 arm: the damaged state with varying degrees of damage (class 0) and the undisturbed state (class 1), as shown in Figs. 5 and 6. In this way, both time-domain and frequency-domain features were prepared, allowing for a comparison of their performance by evaluating the diagnostic effectiveness of the machine learning models.

## Machine learning analysis

Referring to Fig. 13, four artificial intelligence models as K-Nearest Neighbors (KNN), Decision Trees (DT), Random Forest (RF), and Artificial Neural Networks (ANN) were successfully applied to the obtained statistical features mentioned in the previous subsection, arranged in form of tabular data.

The KNN model^69,70^ was chosen for its simplicity and intuitiveness, making it easy to understand and implement. Additionally, it is effective for small datasets with well-separated data containing complex nonlinear correlations. The DT model^71,72^ is also characterized by its ease of interpretability and ability to identify nonlinear relationships in the data. It features a short training and prediction time, even for large datasets. The RF model^73,74^, an extension of the DT model, reduces the risk of overfitting while maintaining high or even greater accuracy, even in the presence of noisy data. Artificial Neural Networks (ANN)^75,76^ have the ability to detect complex, subtle, and intricate patterns and correlations in the data while being a flexible and scalable family of algorithms.

These models play a crucial role in UAV motor damage diagnostics by enabling pattern recognition, anomaly detection, and classification of damage severity, thereby improving reliability. All models were implemented using the appropriate Scikit-learn libraries in Python^77,78^, without changing the default model parameters. The architecture of the deep neural network consisted of 6 layers with the following number of neurons: 40, 256, 128, 64, 32, and 1 in the output layer. Each layer was followed by a batch normalization layer and a dropout layer with a rate of 0.2. The ReLU activation function was used for all layers except the classification layer, where a sigmoid function was applied. The ADAM algorithm with a learning rate of 0.001 was used as the optimization function.

As the evaluation metric for the quality of each model, the accuracy metric (ACC) was adopted. Accuracy is defined as the percentage of all correct predictions relative to all cases. It indicates how well the model classifies both positive and negative cases. Accuracy is a fundamental metric for assessing model quality, particularly in the context of balanced classes. The input datasets consisted equally of samples from motors without damage and engines with damage, making it a binary classification problem where accuracy can be sufficient. After the application of ML models was completed, the accuracy of each prepared model was evaluated. For each statistical feature, 80% of the obtained data was used for training, while the remaining 20% was allocated for validation.

## Results discussion

**Fig. 14:**
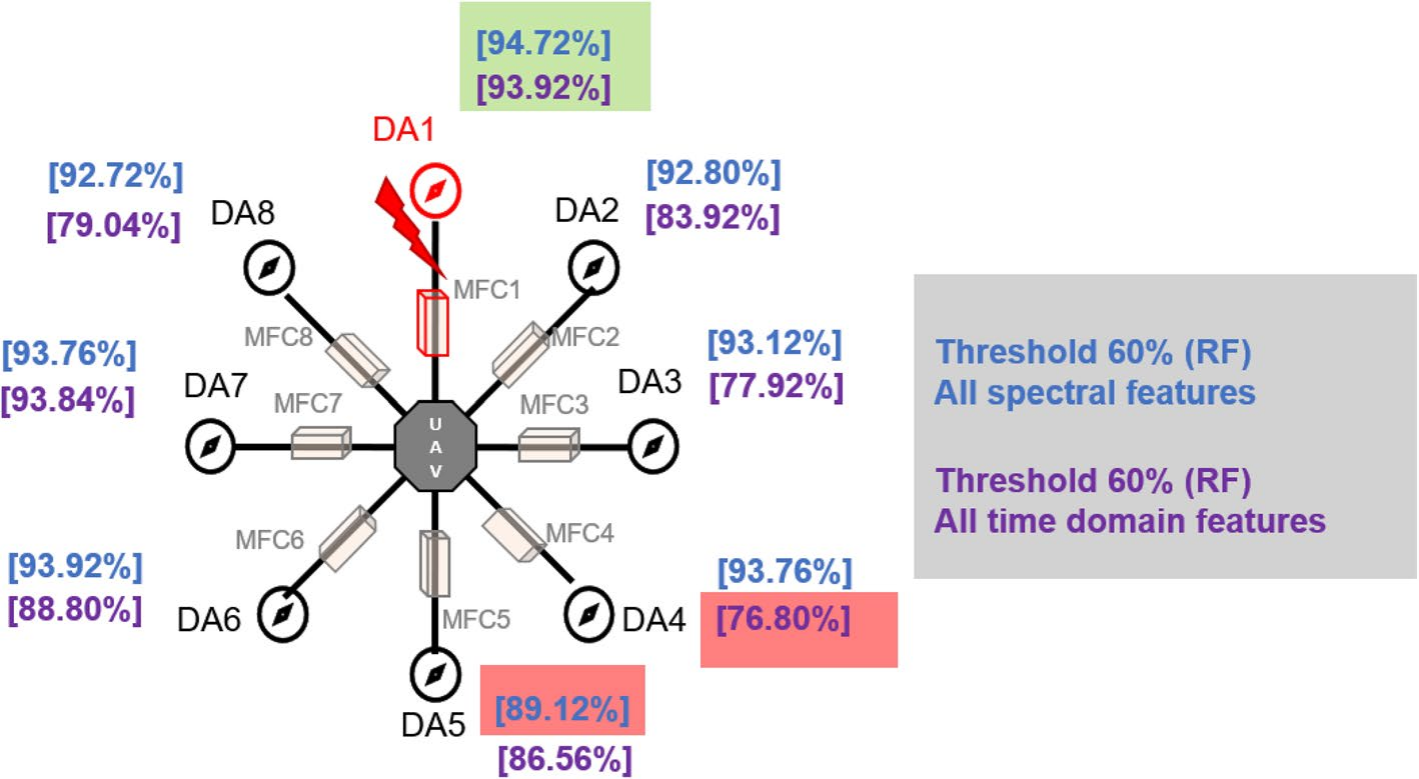
Results of accuracy distribution along the drone’s arms for all time-domain features in Ground Mode.

**Fig. 15:**
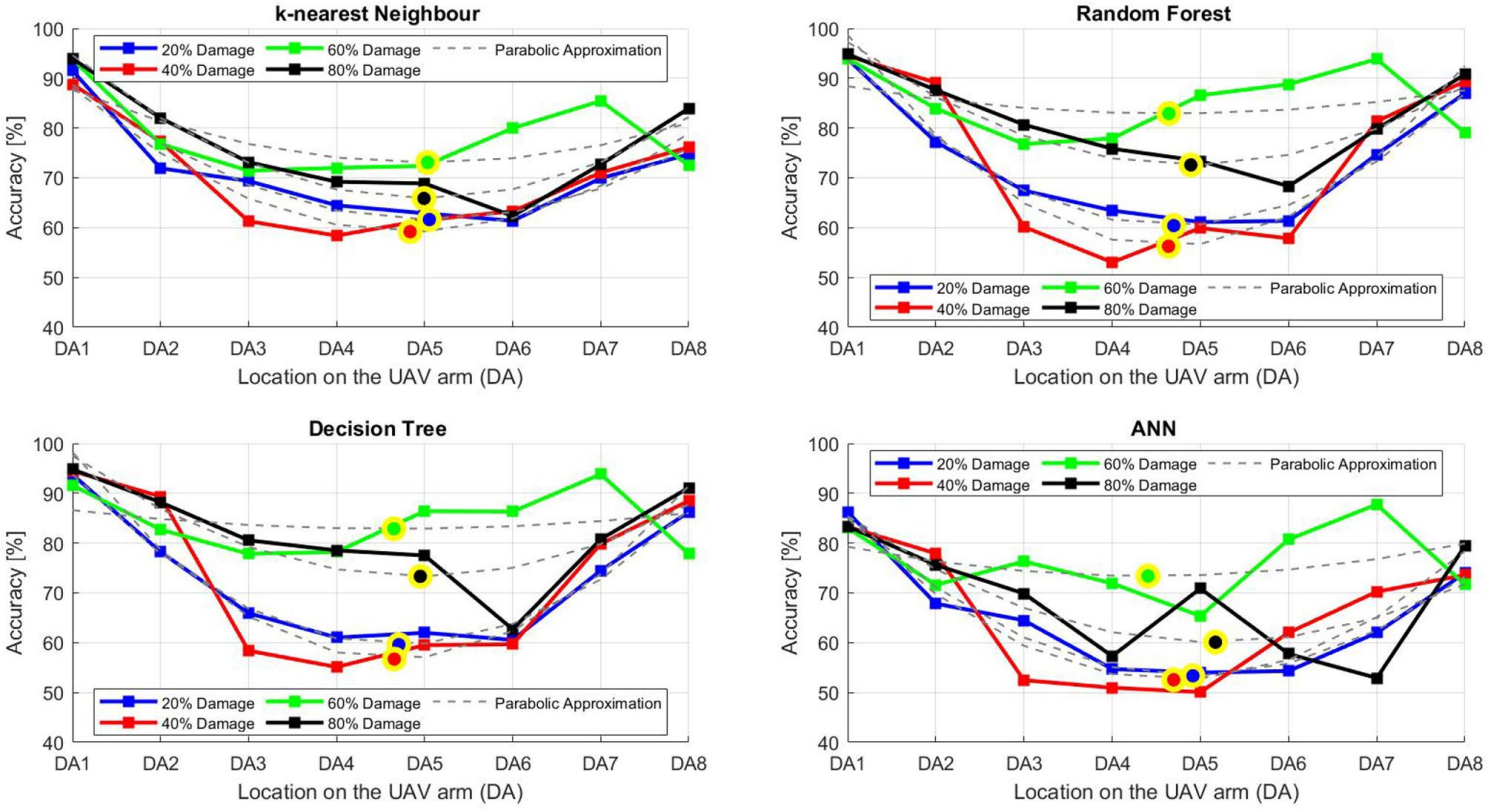
Spider Plot of Accuracy Distribution along the Drone’s Arms for all time-domain features in Ground Mode.

**Fig. 16:**
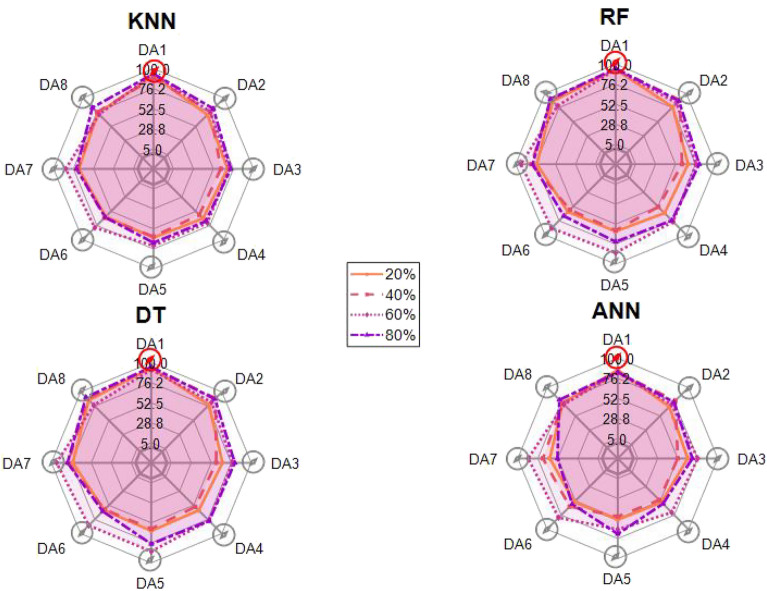
Results of accuracy distribution along the drone’s arms for only RMS feature in Ground Mode.

**Fig. 17:**
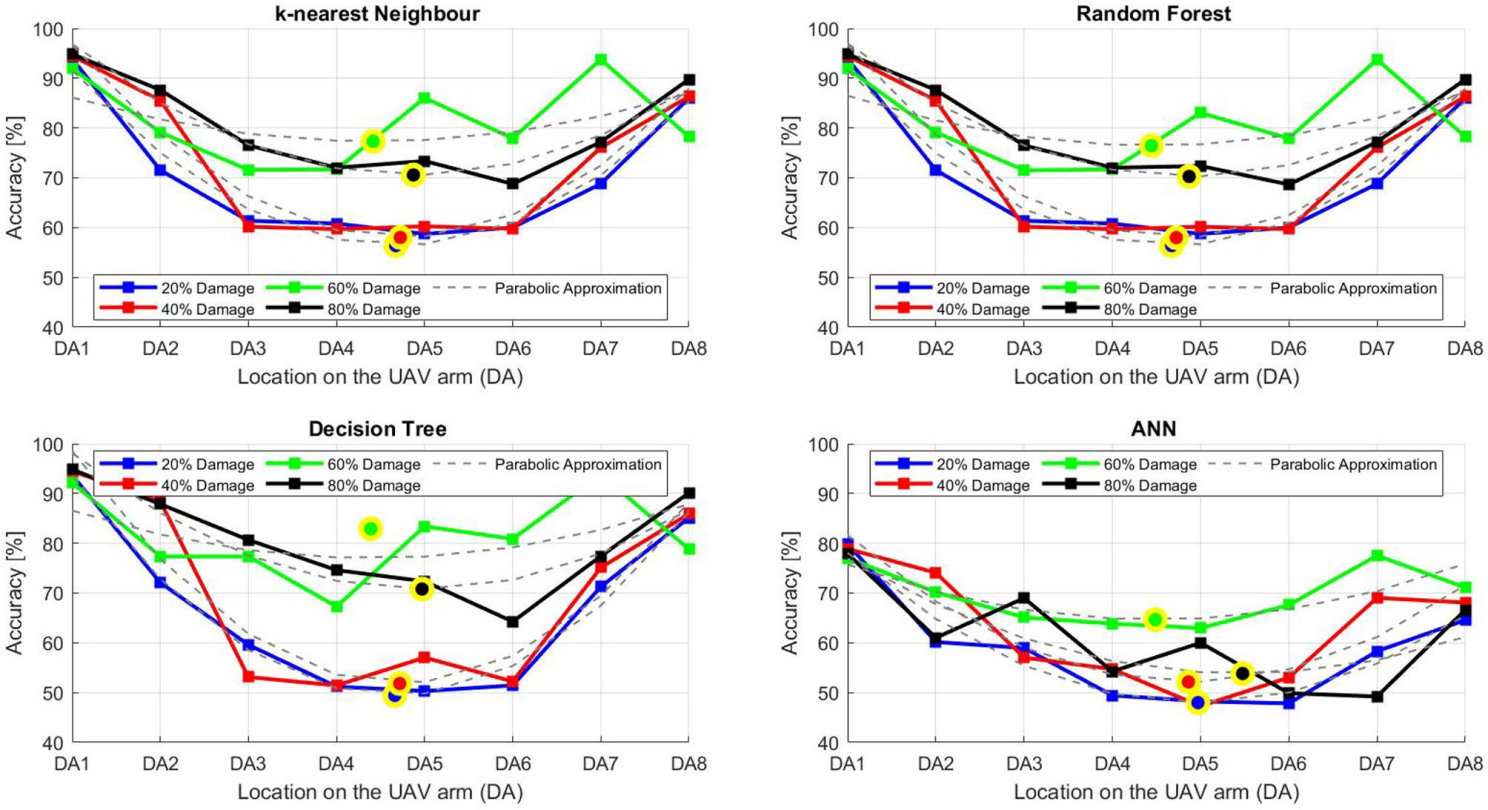
Spider Plot of Accuracy Distribution along the Drone’s Arms for the RMS Feature in Ground Mode.

**Fig. 18:**
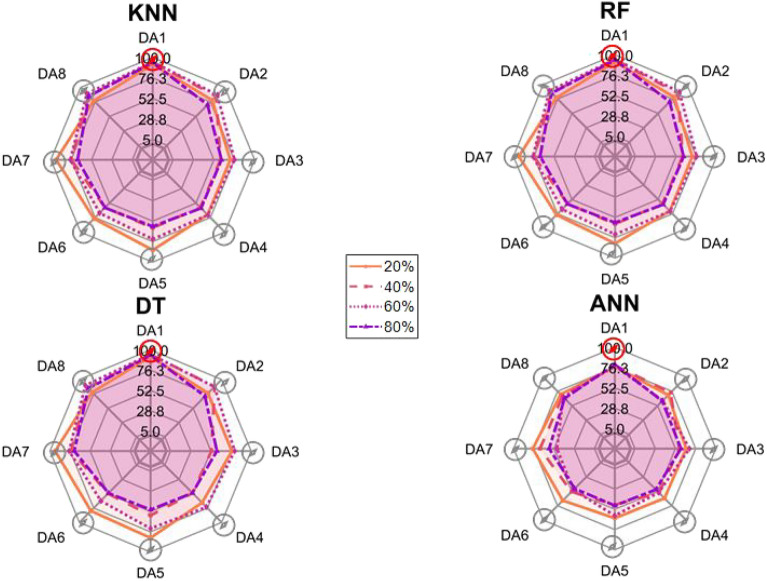
Results of accuracy distribution along the drone’s arms for all frequency-domain features in Ground Mode.

**Fig. 19:**
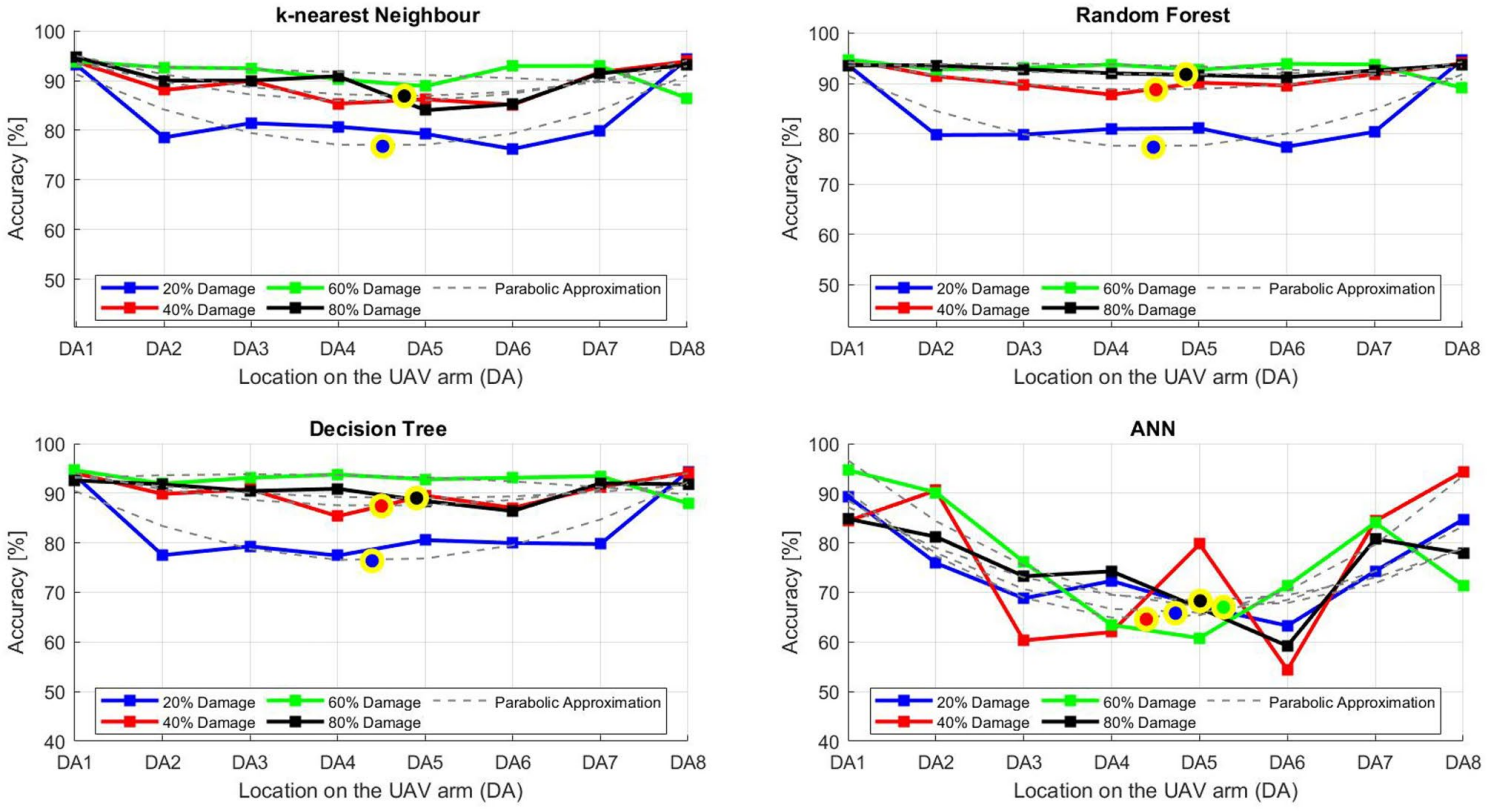
Spider Plot of Accuracy Distribution along the Drone’s Arms for all frequency-domain features in Ground Mode.

**Fig. 20:**
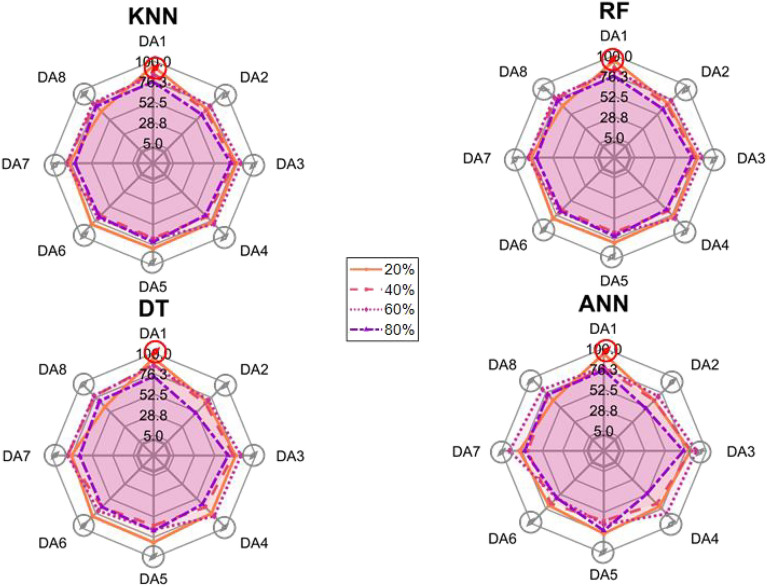
Results of accuracy distribution along the drone’s arms for only Spectral Centroid feature in Ground Mode.

**Fig. 21:**
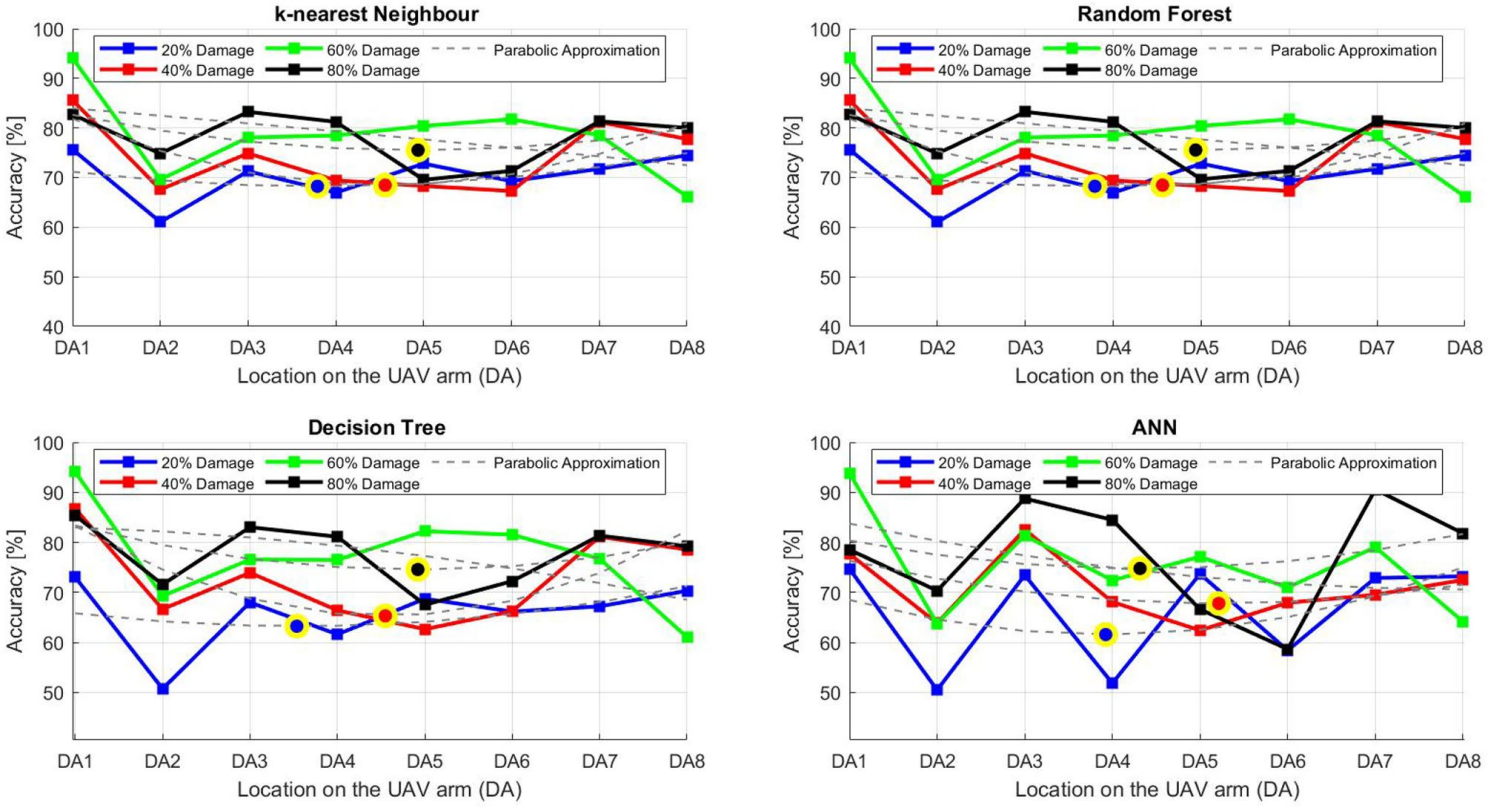
Spider Plot of Accuracy Distribution along the Drone’s Arms for the Spectral Centroid Feature in Ground Mode.

**Fig. 22:**
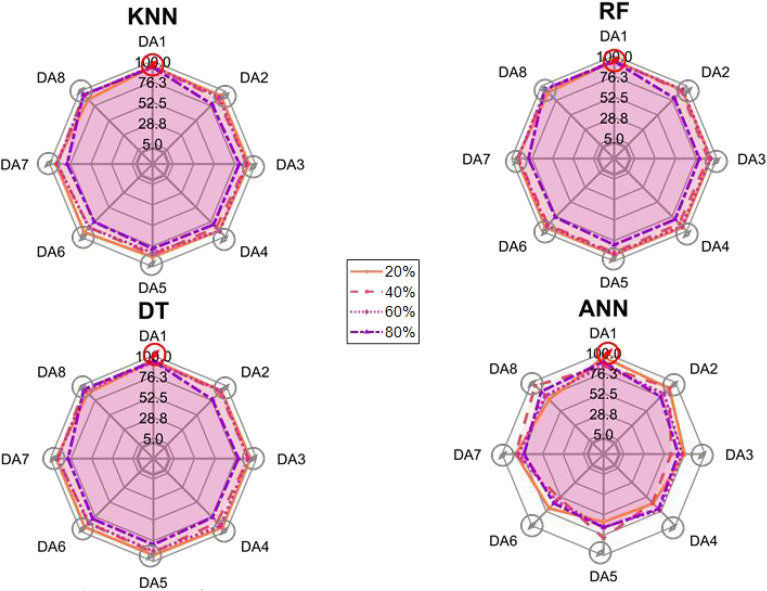
Results of accuracy distribution along the drone’s arms for all time-domain features in Fly Mode.

**Fig. 23:**
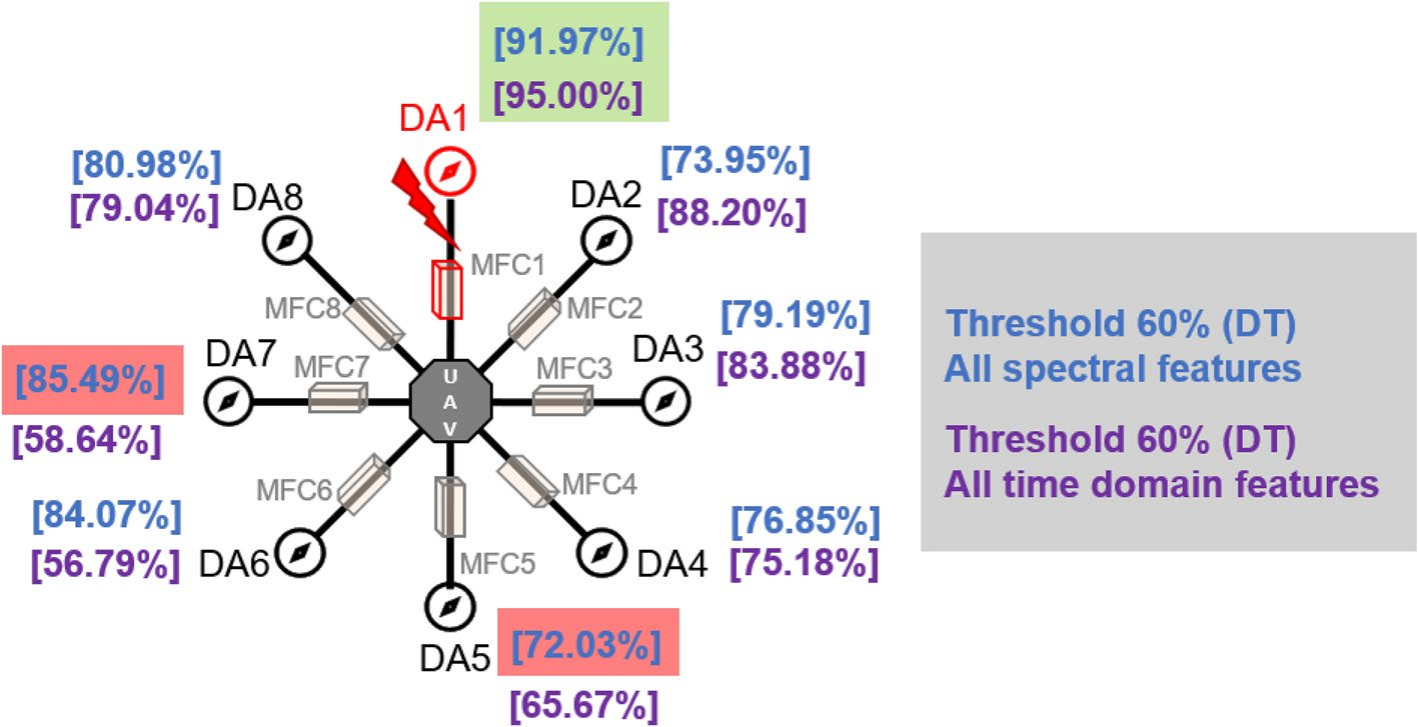
Spider Plot of Accuracy Distribution along the Drone’s Arms for all time-domain features in Fly Mode.

**Fig. 24:**
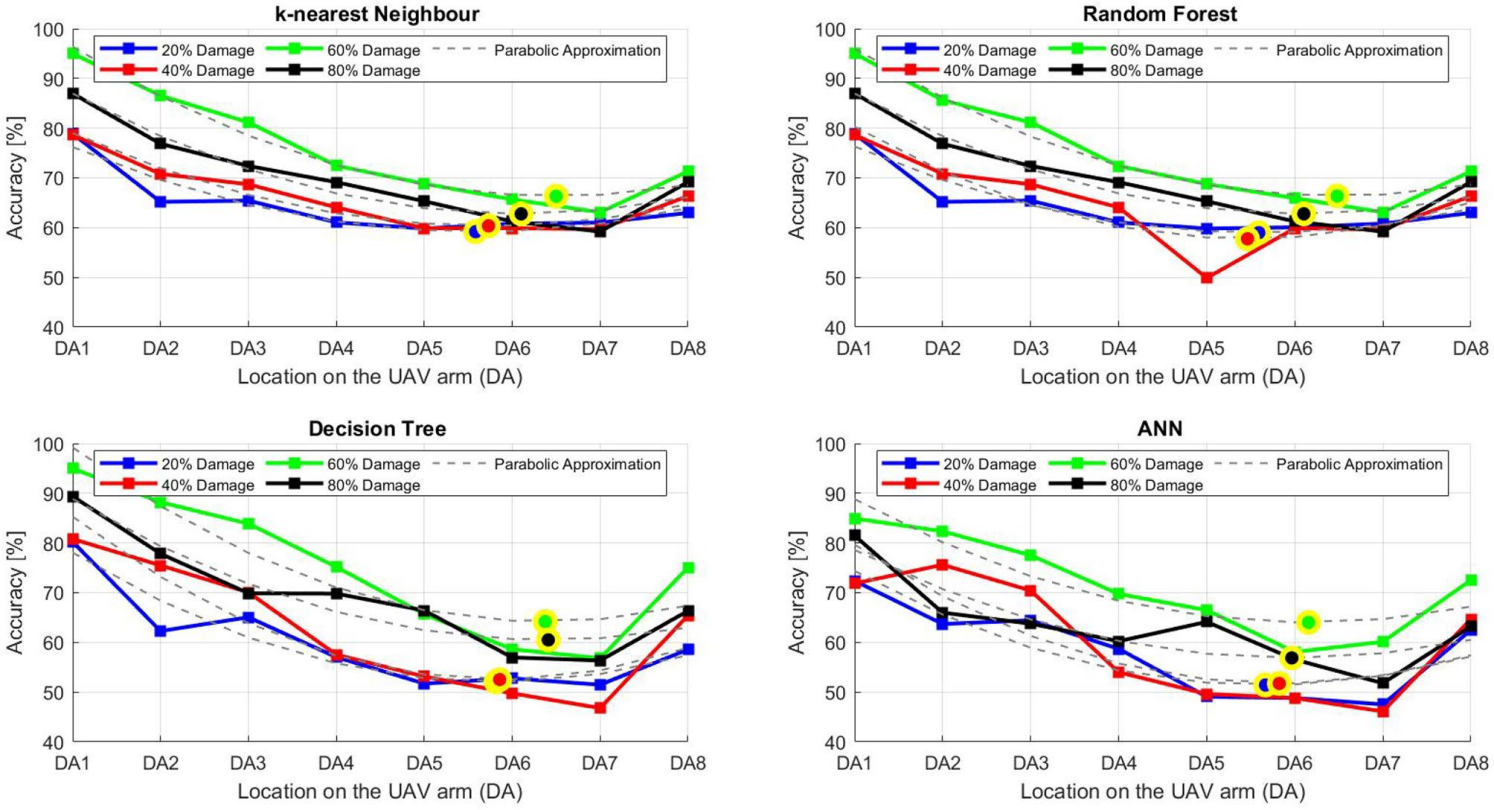
Results of accuracy distribution along the drone’s arms for only RMS feature in Fly Mode.

**Fig. 25:**
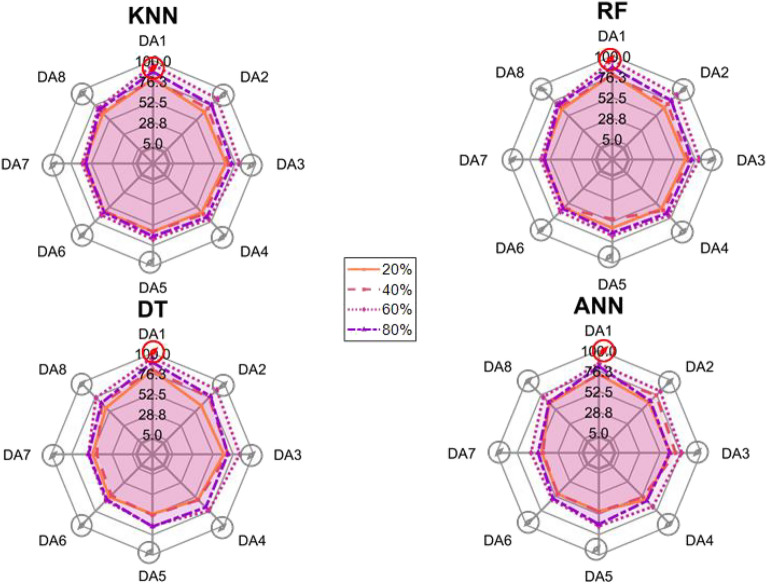
Spider Plot of Accuracy Distribution along the Drone’s Arms for the RMS Feature in Fly Mode.

**Fig. 26:**
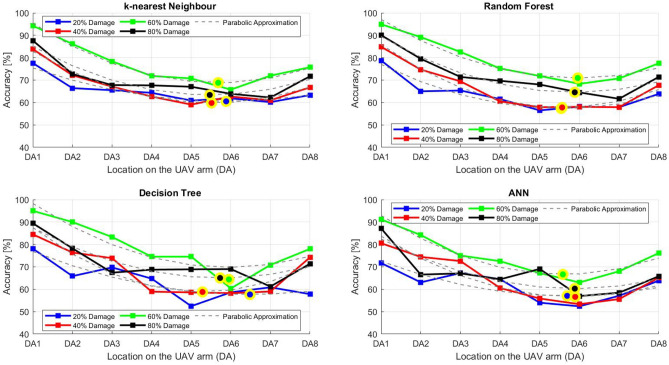
Results of accuracy distribution along the drone’s arms for all frequency-domain features in Fly Mode.

**Fig. 27:**
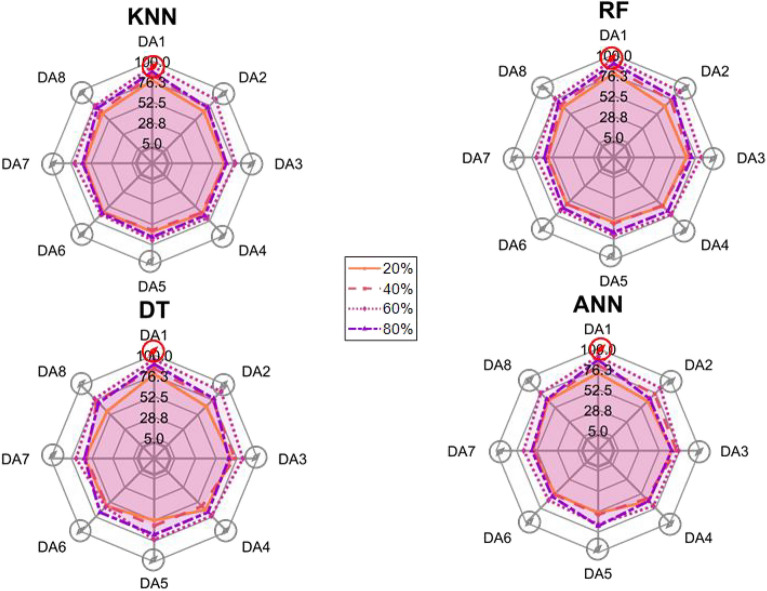
Spider Plot of Accuracy Distribution along the Drone’s Arms for all frequency-domain features in Fly Mode.

**Fig. 28:**
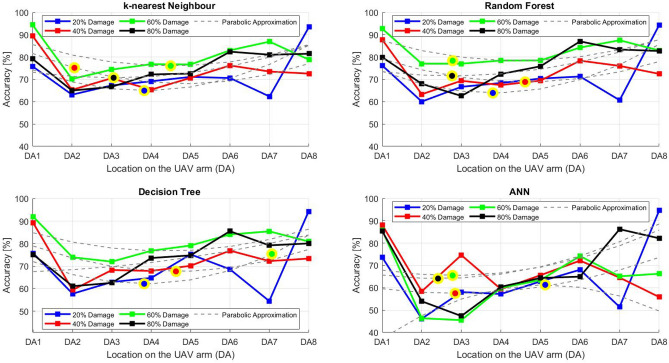
Results of accuracy distribution along the drone’s arms for only Spectral Centroid feature in Fly Mode.

**Fig. 29:**
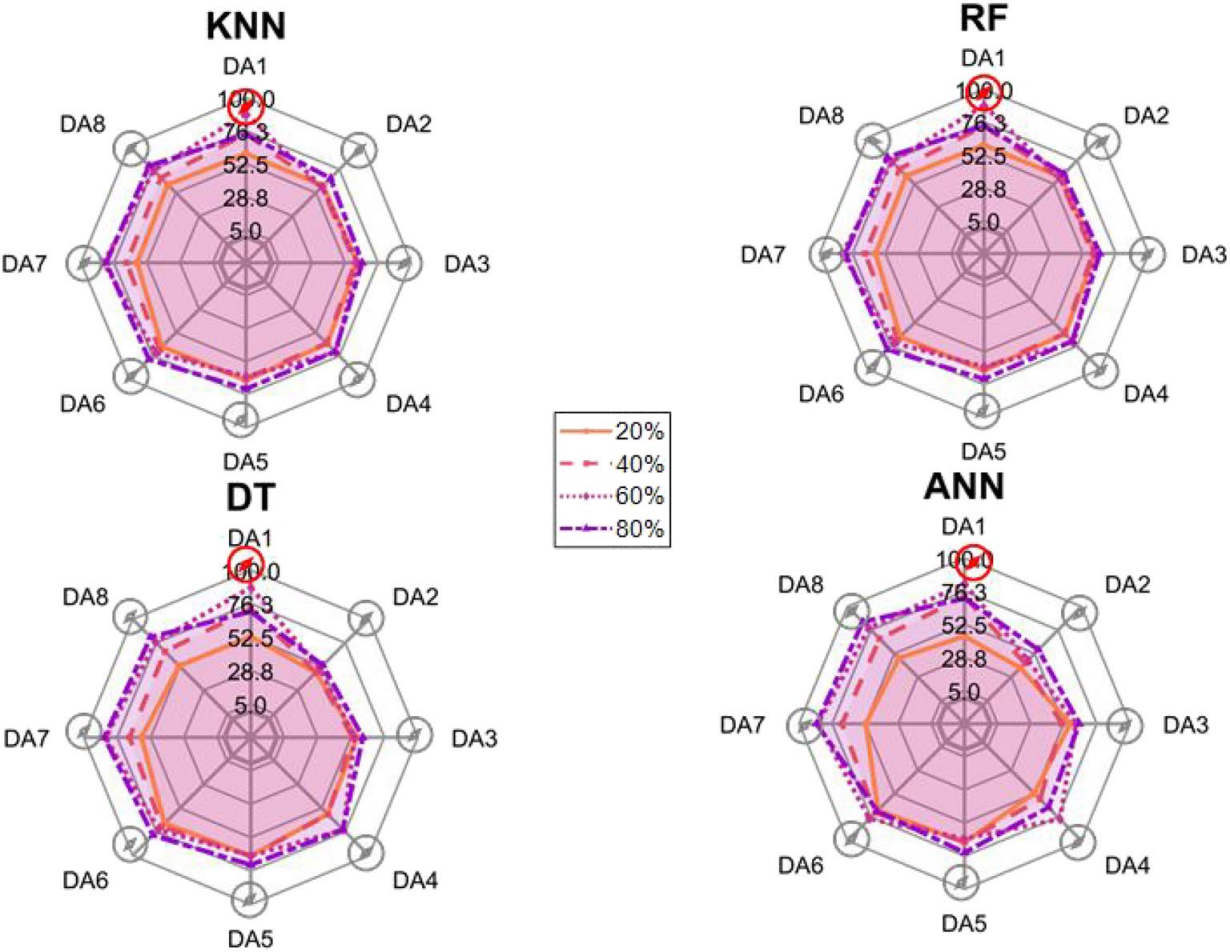
Spider Plot of Accuracy Distribution along the Drone’s arms for the spectral centroid feature in fly mode.

**Fig. 30:**
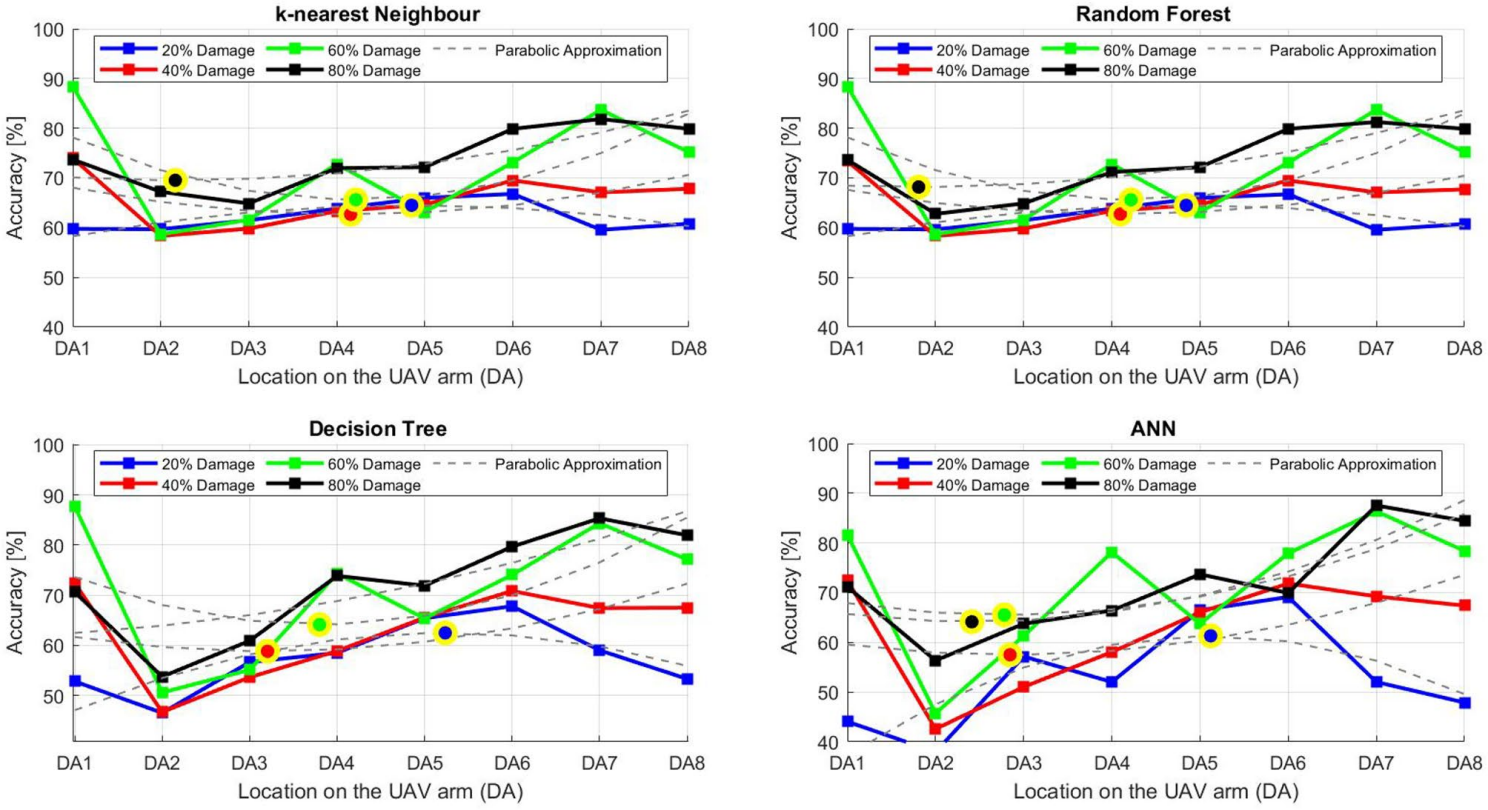
Diagram of a drone with marked arms and machine learning results (Results based on all Time Domain Features, all Spectral Features for Different Damage Levels: Classification Performance of KNN, DT, RF, and ANN Methods (Ground Mode), case Threshold = 60%, Random Forest).

**Fig. 31:**
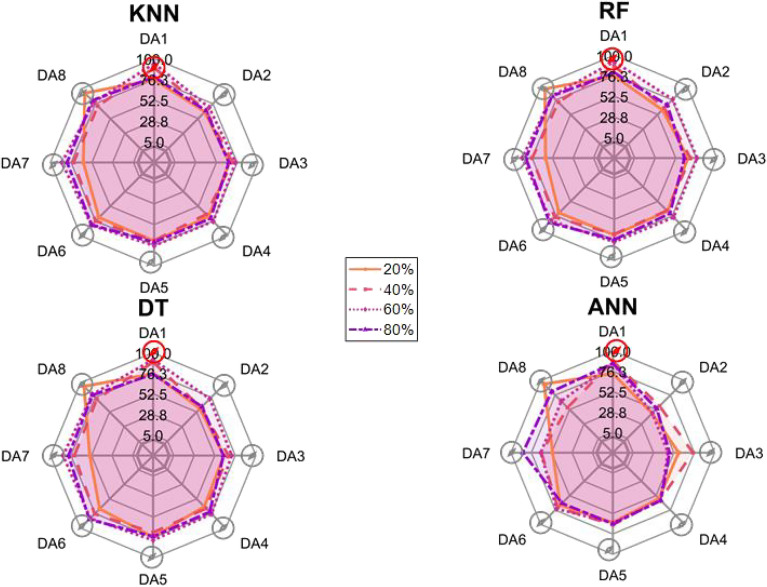
Diagram of a drone with marked arms and machine learning results (Results based on all Time Domain Features, all Spectral Features for Different Damage Levels: Classification Performance of KNN, DT, RF, and ANN Methods (Fly Mode), case Threshold = 60%.

After the experiment and machine learning analysis were conducted, the next step was the analysis of the results obtained for the studied ML models. The results for both Ground Mode and Fly Mode were presented in the form of spider plots separating the results obtained on each drone’s arm (DA1-DA8) across the four studied ML models and by the specific threshold of the damage. In addition to spider plots, the results were also presented in the graphical form of parabolic approximation finding the minima of classification for each load case (see Figs. 14, 15, 16, 17, 18, 19, 20, 21, 22, 23, 24, 25, 26, 27, 28 and 29). The motivation for applying the parabolic approximation to the results was to illustrate their distribution relative to the drone’s arms. Exemplary results showing how the accuracy corresponds to a specific drone arm are presented in Fig. 30 (one load case from Ground Mode) and Fig. 31 (one case from Fly Mode), and are related to the marking presented in Fig.1b. The comparison between Ground and Fly Modes was conducted as follows for 4 feature selections, i.e.: all time-domain features, only RMS feature, only Spectral Centroid feature, all frequency-domain features. The classification performance achieved using time-domain features was generally lower compared to frequency-domain features, particularly on drone arms more distant from the damaged motor. This can be explained by structural damping and signal attenuation during vibration propagation, which reduce the amplitude and distinctiveness of time-domain characteristics such as RMS or peak-to-peak values. These features become less reliable at capturing localized damage once the signal spreads across the UAV frame. In contrast, frequency-based features retain more stable patterns, as spectral characteristics are less sensitive to distance-related energy loss and exhibit higher resilience to noise−especially in Fly Mode scenarios where more complex dynamics are involved. This trend is further explained by the spectral properties of the signals obtained during the tests.

The results presented in Figures 7 - 10 clearly demonstrate the presence of distinct spectral peaks in the power spectral density (PSD) of voltage signals recorded by the piezoelectric sensors, particularly under simulated damage conditions. The method of inducing degradation effectively alters the dynamic response of the UAV arm, resulting in frequency-dependent variations in the voltage signals. While time-domain features are able to capture overall energy fluctuations or statistical dispersion, they are not sufficiently sensitive to the specific spectral shifts introduced by such control-based degradation. Consequently, classifiers based on time-domain descriptors tend to underperform, especially in detecting lower levels of simulated damage. On the other hand, frequency-domain features provide enhanced sensitivity to these variations, as they better capture changes in the energy distribution across specific frequency bands. This is particularly evident in the more pronounced spectral peaks and shifts observed at lower duty cycles. The improved classification accuracy achieved using frequency-based features is thus supported by both the physical modeling of the fault scenario and the empirical spectral analysis.

### Ground mode

The distribution of the first results of time-domain based features is aligned along the parabolic approximation, and the minima for all considered models were found around DA5, that is the opposite to the arm on which the damage was initiated. The general tendency in all results is that the accuracy of the models decreases toward the most opposite arms, while the neighboring to the first one has almost similar accuracy to the main DA1 arm. Comparing the results of accuracy between different levels of damage, it can be stated that it is easier to recognize the damage level for its higher value, i.e. 60% and 80%, nevertheless, the damage recognition on the main arm exceeds over 90% for KNN, RF and DT models, while the ANN classifies the damage group with over 80% accuracy. On the other hand, the smaller values of damage, i.e. 20% and 40% are characterized by strictly with the parabolic alignment of accuracy results, so the propagation of the damage on the arms has a regular tendency, while in case of higher level of damage, some higher accuracy of classification can be also observed on the opposite arms. All results of classification for time-domain features are associated with the spider plot, where all minima of classification were on the opposite arms, i.e. from DA3 to DA6.

The next results of machine learning analysis refer to only one of the time-domain features, i.e. RMS value only. In this case, the distribution of classification accuracy results follows a similar parabolic alignment to that observed in the general time-domain features classification. Similarly, the minima of parabolic approximation for all studied cases and models were found around the most opposite arm, i.e. DA5. The accuracy of the models shows a decreasing trend towards the most opposite arms, while the adjacent arms to the main DA1 maintain accuracy levels close to those observed on the main one. Again, comparing the results across different levels of damage, it is evident that higher damage levels (60% and 80%) are more easily recognized. However, even for lower damage levels (20% and 40%), the accuracy results align strictly with the parabolic trend, reinforcing the systematic propagation of damage along the arms. For the highest damage levels, some deviations from this trend were noted, where classification accuracy occasionally increased on the opposite arms. Regarding specific models, KNN, RF, and DT achieve over 90% accuracy for detecting damage on the main arm, while as above, ANN provides an accuracy exceeding 80%. These trends remain consistent with the general time-domain feature analysis. The classification results for the RMS-only feature are detailed in the spider plot, where the minima of classification accuracy were consistently observed on the opposite arms DA4 to DA6.

For frequency-domain features, the distribution of classification accuracy results differs from the strict parabolic alignment observed for time-domain features. While the minima of classification accuracy are still found around the most opposite arm (DA5) for some models, for all models−i.e. KNN, RF, and DT−the classification results follow a parabolic function, showing a more systematic pattern. However, for the 60% damage level, the minima were not identified for the three models, but the damage level was recognized on each arm with over 90% accuracy. The overall classification accuracy for frequency-domain features is notably high. For the three models, classification accuracy exceeds 80% across all arms, except for the ANN model, which shows lower performance in some cases. In terms of damage level differentiation, the results remain consistent with previous findings: higher damage levels (40%, 60%, and 80%) achieve accuracy levels close to 90%, making them more distinguishable compared to the lowest damage level (20%). Despite these variations, a general gradation in recognition accuracy is still observable. While most of the classification minima for frequency-domain features remain located around the opposite arm (close to DA5), for the 60% damage level, the minima of parabolic approximation fall outside the range. The classification results are summarized in the spider plot, reinforcing the overall trend that frequency-domain features allow for robust differentiation of severe damage cases, though certain models deviate from the expected parabolic alignment.

For the Spectral Centroid feature, the distribution of classification accuracy results differs from the strict parabolic alignment observed for time-domain features, including RMS. While the minima of classification accuracy are still found around the most opposite arm (DA5), the results do not follow a strictly parabolic trend. Instead, there is a more irregular distribution, though a general gradation in recognition accuracy can still be observed. In terms of damage level differentiation, the results remain consistent with previous findings: higher damage levels (60% and 80%) are more easily recognized than lower ones (20% and 40%). However, unlike RMS, the neighboring arms do not consistently match the levels observed on DA1, indicating a less systematic propagation pattern. Regarding classification performance, the highest accuracy results were observed for the most severe damage cases (around 80%), while the lowest accuracy was found for the smallest damage levels (around 20%). Despite these variations, half of the classification minima were still located on the opposite arms (close to the DA5), but for damage 20% and 60%, they were found on the neighboring arms as detailed in the spider plot.

Table 2, the results of the identified classification accuracy minima for each model and each damage level of the electric motor is summarized. In the case of the Ground Mode, the majority of numerical classification accuracy minima were concentrated on the opposite arms of the drone, specifically DA4, DA5, and DA6. These arms account for a total of 53 out of 64 possible cases where minimum accuracy was observed. This strong localization of classification minima suggests that time-domain and frequency-domain indicators effectively capture the spatial distribution of motor damage effects. Consequently, their utilization in the development of automated diagnostic models for assessing the severity of motor damage in drones appears to be well-founded. To contextualize our findings, it is important to note that prior studies using vibration-based UAV diagnostics typically focused on isolated motor faults without examining propagation effects across the airframe. For instance, in^40^, drone motor faults classified from current signals yielded accuracies between 85%–92% using neural networks, while^33^ achieved 88% using AI on onboard accelerometers. In contrast, our models reached 93–94% accuracy on the directly damaged arm and over 80% on adjacent arms using piezo-based structural measurements, offering richer insight into systemic effects. Furthermore, none of the above methods investigated spatial damage propagation or validated classification performance arm-by-arm in both ground and flight conditions.

Although no directly comparable public benchmark exists for UAV structural damage propagation, our method demonstrates superior spatial resolution and interpretability. The parabolic accuracy profiles along the drone’s arms illustrate clear and predictable damage patterns, which represent a novel contribution compared to black-box classifiers applied to aggregated data in earlier works.

**Table 2:** Results of maximal and minimal values of classification found on each drone arm (DA), for the Ground Mode.

Drone arm	DA1	DA2	DA3	DA4	DA5	DA6	DA7	DA8
Number of maxima	64	0	0	0	0	0	0	0
Number of minima	0	5	3	11	19	23	1	3

### Fly tests

For Fly Mode, the distribution of the initial classification results based on time-domain features follows a parabolic approximation, with all classification accuracy minima located near DA6. This suggests that the most pronounced decrease in accuracy occurs on one of the opposite arms relative to the damage initiation point. The general trend indicates that classification accuracy decreases toward the most opposite arms, whereas the neighboring arms to the damage initiation point exhibit accuracy levels slightly lower than those observed on the main one arm. When comparing accuracy across different damage levels, it is evident that higher damage levels (60% and 80%) are more easily recognized than lower ones (20% and 40%). Notably, on the main arm (DA1), the classification accuracy exceeds 90% for KNN, RF, and DT models, while the ANN model achieves over 80% accuracy. All classification results for time-domain features in Fly Mode are summarized in Table 3, where all classification accuracy minima were on the opposite arms, particularly around DA5 and DA7. This confirms the systematic propagation of damage across the drone’s structure in Fly Mode, aligning with the observed classification trends.

For the RMS feature in Fly Mode, the distribution of the initial classification results follows a parabolic approximation, with all classification accuracy minima located near DA6 across all models and damage levels. This indicates that the most significant decrease in accuracy occurs on one of the opposite arms relative to the damage initiation point. The general trend shows that classification accuracy decreases toward the most opposite arms, while the neighboring arms to the damaged one exhibit slightly lower accuracy compared to the main one. When comparing classification accuracy across different damage levels, it is evident that lower damage levels (20% and 40%) can be more easily distinguished than higher ones (60% and 80%). This differentiation suggests that the RMS feature effectively captures variations in damage severity. All classification results for the RMS feature in Fly Mode are summarized on the spider plot shown in Fig. 23, where all classification accuracy minima were consistently found around arms DA5 to DA7, reinforcing the systematic propagation pattern of damage within the drone’s structure.

For all frequency-domain features, the distribution of classification accuracy results deviates from the strict parabolic alignment observed for time-domain features. While some classification accuracy minima are still found on the opposite arms, they are also distributed across different arms, leading to a more irregular pattern along the drone’s structure. Unlike time-domain features, where accuracy degradation follows a more systematic trend, the minima of the parabolic approximation occur at varying locations, indicating a less consistent propagation of classification performance. In terms of damage differentiation, the results show that distinguishing between different damage levels is more challenging compared to time-domain features. Although higher damage levels (60% and 80%) still tend to achieve better recognition than lower ones (20% and 40%), the separation between damage levels is not as distinct. Classification performance remains relatively high for severe damage cases, but the lack of a clear trend in accuracy distribution suggests a more complex relationship between frequency-domain features and damage propagation. Despite these inconsistencies, frequency-domain features still offer valuable insights for damage classification in Fly Mode. The classification accuracy results are summarized in Fig. 25 in the form of a spider plot, highlighting the dispersed distribution of accuracy minima across both opposite and neighboring arms, as well as the challenges in clearly distinguishing between different damage levels.

For Fly Mode, the distribution of classification accuracy results for the Spectral Centroid feature deviates from the strict parabolic alignment observed for time-domain features. While some classification accuracy minima are still found on the opposite arms, they also appear on neighboring arms, indicating a more irregular distribution along the drone’s structure. Notably, the highest number of classification minima is concentrated around DA2, suggesting a different pattern of accuracy degradation compared to the time-domain features. In terms of damage differentiation, the results remain consistent with previous findings: higher damage levels (60% and 80%) are more easily recognized than lower ones (20% and 40%). However, unlike RMS, where accuracy trends follow a more systematic propagation pattern, the Spectral Centroid results exhibit a more dispersed distribution. Classification performance remains high for severe damage cases, while the lowest accuracy was observed for the smallest damage levels. Despite these irregularities, damage levels can be effectively distinguished from one another, demonstrating the potential of frequency-domain features for damage classification in Fly Mode. The classification accuracy results are summarized in Fig. 27, highlighting the spread of accuracy minima across both opposite and neighboring arms.

**Table 3:** Results of maximal and minimal values of classification found on each drone arm (DA), for the Fly Mode.

Drone Arm	DA1	DA2	DA3	DA4	DA5	DA6	DA7	DA8
Number of maxima	64	0	0	0	0	0	0	0
Number of minima	0	25	3	0	10	8	18	0

In Table 3, the summary of results of the identified classification accuracy minima for each model and each damage level of the electric motor is presented. In the case of Fly Mode, the majority of numerical classification accuracy minima are distributed across the following arms: DA2, DA5, DA6, and DA7. Specifically, DA2 accounts for 25 minima, while DA5, DA6, and DA7 collectively account for 36 out of 64 considered configurations where minimum accuracy was observed. This distribution indicates a more dispersed pattern of classification accuracy degradation compared to Ground Mode. Nevertheless, the concentration of minima on specific arms suggests that time-domain and frequency-domain indicators effectively capture the spatial distribution of motor damage effects in Fly Mode. Consequently, their application in developing automated diagnostic models for assessing the severity of motor damage in drones remains well-founded.

The observed parabolic distribution of classification accuracy across the drone’s arms is significant−it suggests that vibration signals induced by localized motor faults propagate structurally through the UAV frame in a systematic manner. This finding implies that damage is not limited to the failing component but also affects the surrounding mechanical structure, a point rarely addressed in prior UAV fault diagnostics.

The consistent decrease in accuracy with increasing distance from the damaged motor (DA1) validates the use of spatially distributed sensors for assessing fault severity and propagation. The higher accuracy achieved for frequency-domain features, especially on distant arms, indicates that spectral components of the vibration signal may carry more robust information about low-amplitude or secondary responses induced by the fault. In contrast, time-domain features are more sensitive to localized and high-amplitude signals, which explains their stronger performance near the damage source.

Moreover, the results demonstrate that for higher damage levels (60–80%), structural responses are detectable even on opposite arms, which could inform redundancy or health monitoring strategies in UAV design. This supports the integration of structural sensors not only near critical components but also in locations that can capture systemic behavior under stress.

Unexpectedly, we observed that for lower damage levels (20–40%), classification minima were sharply concentrated on opposite arms in a highly symmetric pattern, indicating a subtle but structured propagation mechanism. This suggests the potential to predict not only the presence but also the trajectory or spread of a developing fault, which is valuable for predictive maintenance systems.

These findings emphasize that fault detection methods should not treat the UAV as a set of isolated components but as a dynamic, interconnected structure. Incorporating both time- and frequency-domain features from spatially distributed sensors enables a more holistic diagnostic perspective. The methodology presented here can serve as a foundation for next-generation UAV health monitoring systems with improved sensitivity, coverage, and interpretability.

## Summary

This study presents and experimentally validates a novel diagnostic framework for detecting drive system faults in unmanned aerial vehicles (UAVs) using piezoelectric sensors and machine learning. Sensors were mounted on each arm of an octocopter, and controlled motor degradation was simulated through variations in the PWM duty cycle. Voltage signals were acquired in both Ground and Fly Modes, and short-time sliding windows were used to extract statistical features in both the time and frequency domains. These features served as input to machine learning classifiers (Random Forest, k-Nearest Neighbors, and Decision Tree) to detect motor damage and analyze its propagation across the UAV structure. The approach achieved high classification accuracy, revealed distinct spatial patterns of damage propagation, and provides new insights to UAV health monitoring systems with distributed sensing. The evaluation of both time- and frequency-based features revealed the latter to be more robust in detecting structural damage propagation, which was further supported by FFT spectral analysis.

The key findings of the study are summarized below:

- **Damage propagation:** Time-domain features showed a more systematic classification pattern, while frequency-domain features had a more irregular distribution of accuracy minima.
- **Classification accuracy:** Higher damage levels (60% and 80%) were more easily distinguished than lower levels (20% and 40%).
- **Ground mode:** Classification minima were mainly found on opposite arms (DA4, DA5, DA6), indicating clear damage detection.
- **Fly mode:** Classification minima were more dispersed, with notable occurrences on DA2 and DA5 to DA7, reflecting more complex damage propagation.
- **Accuracy trends:** The highest accuracy was achieved for the arm where the motor failure was induced, while the opposite arm showed the lowest performance.
- **Machine learning models:** k-Nearest Neighbors (KNN), Random Forest (RF), Decision Tree (DT), and Artificial Neural Network (ANN) models demonstrated effective classification, with RF and KNN showing strong performance across most damage levels.

Compared to existing methods, which typically report classification accuracies in the range of 85–92% for isolated motor faults, the proposed approach achieved comparable or higher diagnostic accuracy, along with the added benefit of structural propagation insight. This enables not only fault detection but also interference about affected regions across the UAV structure, supporting more robust predictive maintenance strategies.

Future work will focus on implementing the diagnostic algorithm in real-time using embedded systems such as microcontrollers or lightweight edge-AI platforms. Additional research will integrate complementary sensors (e.g., accelerometers and gyroscopes) to improve signal diversity and fault resolution. The method will also be validated under varying flight conditions, such as wind disturbances, payload shifts, and different UAV architectures. Finally, a larger dataset including additional fault types and degradation trajectories will be developed to ensure model robustness in practical UAV missions.

## Data Availability

Data will be made available on request. Correspondence and requests for materials should be addressed to A. Koszewnik.
